# Supplementary material for: De novo assembly, annotation, marker discovery, and genetic diversity of the Stipa breviflora Griseb. (Poaceae) response to grazing
Source: PLoS One. 2020 Dec 22;15(12):e0244222. doi: 10.1371/journal.pone.0244222 (PMC7755183; doi:10.1371/journal.pone.0244222)
Supplement: S1 Table — (DOCX) [file pone.0244222.s001.docx]

**S1 Table. Twenty-one candidate microsatellite loci for *S. breviflora***

| **Gene ID** | **Primer sequence (5'→3‘)** |
| --- | --- |
| CL15497.Contig7_All | ATCGTCAAACTCCACCTAATCAA |
|  | AGGCAATATTGGCAACTCACTC |
| Unigene29061_All | ATATCGAGACGGAGGGAGTATTT |
|  | CCATGTACGCTAGAGCCTAGAAA |
| CL15360.Contig13_All | CCATCTTGGCATCTTCTGCT |
|  | TATGTAATGAACGGGAAGAAGGA |
| Unigene22811_All | CCAGATATGGATTAAACAGAAGC |
|  | CACAAAATCGACGAAGGAACC |
| Unigene12747_All | GAGCGATGCAACGATTATATAGG |
|  | CATCGTGAAGTGATAAGAAGCCT |
| CL749.Contig17_All | CCTGATAAGTGGTTTGACCTCAG |
|  | CCTTATGGTTGAAAAGTGCAATC |
| CL9938.Contig1_All | CTACGCTTTAGTGTTGGGTTCAG |
|  | AAGGCTACGGGAACTCCTTC |
| CL616.Contig34_All | AATCAGCTCGTCGGTATTTGAT |
|  | AGAGGGGAAGAACGAAATATCTG |
| CL5620.Contig1_All | CTACTACTTAGGCGGGATGACAA |
|  | CACGTGATCATTGTTGTCTTGTT |
| CL1837.Contig15_All | GGGGAGTTGGACTTGGTAGTG |
|  | CTTAACCTCCCTTCTCCACCTT |
| CL966.Contig12_All | CACTGGGTTCTCTTCGTCTCC |
|  | TCTCCTCCTGCATCTTTCGTC |
| CL966.Contig15_All | CACCGGATGCAAAGAAACCG |
|  | CCTCCTGCATCTTTCGTCCTC |
| Unigene21574_All | TATGTAGGCCGCGAGAGAGA |
|  | AACCCCCATCAGGAGTGGAA |
| CL14453.Contig1_All | GAGGAAGCGTCGATCGTGAC |
|  | TGTCCACTTTCTGCTCCACG |
| CL5285.Contig5_All | TATAGGCAGTGGGGAGACGA |
|  | AGCTTCGAGGGATGAGGAGA |
| CL1138.Contig10_All | TCCGGCAGGGAACGAATTG |
|  | CTTTGTGTGGCCTCCGTCTC |
| 2CL616.Contig34_All | AGCTCAGGGCTGCGTCTC |
|  | CGTCTTCTCGATCTCGTGG |
| CL15360.Contig13_All | CCTTCGCCATCTTGGCATCT |
|  | ACGAACAGAGAACCTGTCGG |
| CL9938.Contig1_All | CGCTTTAGTGTTGGGTTCAGC |
|  | GGAGTGTGTCGGGACCAAAG |
| Unigene5857_All | CGTACGTACCGCACCAAGT |
|  | AGGTCATACGGGACGAGGAC |
| CL12883.Contig1_All | CGCCGCACGAGAGATCC |
|  | GCTGCTTCGATTCGCTTCTC |
