# Supplementary material for: De novo assembly, annotation, marker discovery, and genetic diversity of the Stipa breviflora Griseb. (Poaceae) response to grazing
Source: PLoS One. 2020 Dec 22;15(12):e0244222. doi: 10.1371/journal.pone.0244222 (PMC7755183; doi:10.1371/journal.pone.0244222)
Supplement: Attachment — Submitted filename: PONE-D-20-16248_reviewer-1.pdf [file pone.0244222.s003.pdf]

PLOS ONE

De novo assembly, annotation, marker discovery, and genetic diversity of the Stipa breviflora Griseb. (Poaceae) response to grazing

--Manuscript Draft--

**Manuscript Number:**	PONE-D-20-16248
**Article Type:**	Research Article
**Full Title:**	De novo assembly, annotation, marker discovery, and genetic diversity of the Stipa breviflora Griseb. (Poaceae) response to grazing
**Short Title:**	Transcriptome response and genetic diversity of S. breviflora to grazing
**Corresponding Author:**	Jianming Niu<br>School of Ecology and Environment<br>Hohhot, CHINA
**Keywords:**	de novo assembly; annotation; genetic diversity; microsatellite markers; grazing; S. breviflora
**Abstract:**	Grassland is one of the most widely-distributed ecosystems on Earth and provides a variety of ecosystem services. Grasslands, however, currently suffer from severe degradation induced by human activities and climate change. In the present study, we explored the transcriptome response of Stipa breviflora, a dominant species in the desert steppe, to grazing through transcriptome sequencing, the development of simple sequence repeat (SSR) markers, and analysis of genetic diversity. De novo assembly produced 111,018 unigenes, of which 88,164 (79.41%) unigenes were annotated. A total of 686 unigenes showed significantly different expression under grazing, including 304 and 382 that were upregulated and downregulated, respectively. These differentially expressed genes (DEGs) were significantly enriched in the “alpha-linolenic acid metabolism” and “plant-pathogen interaction” pathways. Based on transcriptome sequencing data, we developed eight polymorphic molecular markers and investigated the genetic diversity of S. breviflora in grazed and ungrazed sites. We found that a relatively high level of S. breviflora genetic diversity occurred under grazing. This study enriches the knowledge of the genetic background of S. breviflora. The findings of genes that improve resistance to grazing are helpful for the restoration, conservation, and management of desert steppe.
**Order of Authors:**	Dongqing Yan
	Jing Ren
	Jiamei Liu
	Yu Ding
	Jianming Niu
**Additional Information:**	
**Question**	**Response**
**Financial Disclosure**<br><br>Enter a financial disclosure statement that describes the sources of funding for the work included in this submission. Review the submission guidelines for detailed requirements. View published research articles from *PLOS ONE* for specific examples.<br><br>This statement is required for submission and **will appear in the published article** if	This study was supported by Natural Science Foundation of China (NSFC) (31460154, 31860106), and Major Science and Technology Projects of Inner Mongolia Autonomous Region (2019ZD008). JN received the above three awards. The funders had no role in study design, data collection and analysis, decision to publish, or preparation of the manuscript.

Powered by Editorial Manager® and ProduXion Manager® from Aries Systems Corporation

the submission is accepted. Please make sure it is accurate.<br><br>**Unfunded studies**<br>Enter: *The author(s) received no specific funding for this work.*<br><br>**Funded studies**<br>Enter a statement with the following details:<br>• Initials of the authors who received each award<br>• Grant numbers awarded to each author<br>• The full name of each funder<br>• URL of each funder website<br>• Did the sponsors or funders play any role in the study design, data collection and analysis, decision to publish, or preparation of the manuscript?<br>• **NO** - Include this sentence at the end of your statement: *The funders had no role in study design, data collection and analysis, decision to publish, or preparation of the manuscript.*<br>• **YES** - Specify the role(s) played.<br><br>* typeset	
**Competing Interests**<br><br>Use the instructions below to enter a competing interest statement for this submission. On behalf of all authors, disclose any competing interests that could be perceived to bias this work—acknowledging all financial support and any other relevant financial or non-financial competing interests.<br><br>This statement **will appear in the published article** if the submission is accepted. Please make sure it is accurate. View published research articles from *PLOS ONE* for specific examples.	The authors have declared that no competing interests exist.

**NO authors have competing interests**<br><br>Enter: *The authors have declared that no competing interests exist.*<br><br>**Authors with competing interests**<br><br>Enter competing interest details beginning with this statement:<br><br>*I have read the journal's policy and the authors of this manuscript have the following competing interests: [insert competing interests here]*<br><br>* typeset	
**Ethics Statement**<br><br>Enter an ethics statement for this submission. This statement is required if the study involved:<br><br>• Human participants<br>• Human specimens or tissue<br>• Vertebrate animals or cephalopods<br>• Vertebrate embryos or tissues<br>• Field research<br><br>Write "N/A" if the submission does not require an ethics statement.<br><br>General guidance is provided below. Consult the submission guidelines for detailed instructions. **Make sure that all information entered here is included in the Methods section of the manuscript.**	N/A

**Format for specific study types**<br><br>**Human Subject Research (involving human participants and/or tissue)**<br>• Give the name of the institutional review board or ethics committee that approved the study<br>• Include the approval number and/or a statement indicating approval of this research<br>• Indicate the form of consent obtained (written/oral) or the reason that consent was not obtained (e.g. the data were analyzed anonymously)<br><br>**Animal Research (involving vertebrate animals, embryos or tissues)**<br>• Provide the name of the Institutional Animal Care and Use Committee (IACUC) or other relevant ethics board that reviewed the study protocol, and indicate whether they approved this research or granted a formal waiver of ethical approval<br>• Include an approval number if one was obtained<br>• If the study involved *non-human primates*, add *additional details* about animal welfare and steps taken to ameliorate suffering<br>• If anesthesia, euthanasia, or any kind of animal sacrifice is part of the study, include briefly which substances and/or methods were applied<br><br>**Field Research**<br><br>Include the following details if this study involves the collection of plant, animal, or other materials from a natural setting:<br>• Field permit number<br>• Name of the institution or relevant body that granted permission	
**Data Availability**<br><br>Authors are required to make all data underlying the findings described fully available, without restriction, and from the time of publication. PLOS allows rare exceptions to address legal and ethical concerns. See the PLOS Data Policy and FAQ for detailed information.	Yes - all data are fully available without restriction

A Data Availability Statement describing where the data can be found is required at submission. Your answers to this question constitute the Data Availability Statement and **will be published in the article**, if accepted.<br><br>**Important:** Stating 'data available on request from the author' is not sufficient. If your data are only available upon request, select 'No' for the first question and explain your exceptional situation in the text box.<br><br>Do the authors confirm that all data underlying the findings described in their manuscript are fully available without restriction?	
**Describe where the data may be found in full sentences. If you are copying our sample text, replace any instances of XXX with the appropriate details.**<br><br>• If the data are **held or will be held in a public repository**, include URLs, accession numbers or DOIs. If this information will only be available after acceptance, indicate this by ticking the box below. For example: *All XXX files are available from the XXX database (accession number(s) XXX, XXX.)*.<br>• If the data are all contained **within the manuscript and/or Supporting Information files**, enter the following: *All relevant data are within the manuscript and its Supporting Information files.*<br>• If neither of these applies but you are able to provide **details of access elsewhere**, with or without limitations, please do so. For example:<br><br>*Data cannot be shared publicly because of [XXX]. Data are available from the XXX Institutional Data Access / Ethics Committee (contact via XXX) for researchers who meet the criteria for access to confidential data.*<br><br>*The data underlying the results presented in the study are available from (include the name of the third party*	All transcriptome data have been deposited in the NCBI Sequence Read Archive (SRA) with accession number as SRR9903413, SRR9903414, SRR9903415, SRR9903416, SRR9903417, and SRR9903418.

and contact information or URL).<br>• This text is appropriate if the data are owned by a third party and authors do not have permission to share the data.<br><br>* typeset	
Additional data availability information:	Tick here if the URLs/accession numbers/DOIs will be available only after acceptance of the manuscript for publication so that we can ensure their inclusion before publication.

Powered by Editorial Manager® and ProduXion Manager® from Aries Systems Corporation

Manuscript Click here to download Manuscript Manuscript.docx

De novo assembly, annotation, marker discovery, and genetic diversity of the *Stipa breviflora* Griseb. (Poaceae) response to grazing

Dongqing Yan[1¶], Jing Ren[1¶], Jiamei Liu[1], Yu Ding[1], Jianming Niu[1,2,3]*

[1] School of Ecology and Environment, Inner Mongolia University, Hohhot, People's Republic of China

[2] Inner Mongolia Key Laboratory of Grassland Ecology and the Candidate State Key Laboratory of Ministry of Science and Technology, Hohhot, People's Republic of China

[3] Ministry of Education Key Laboratory of Ecology and Resource Use of the Mongolian Plateau, Hohhot, People's Republic of China

* Corresponding author

Email: jmniu2005@163.com (JN)

[¶]These authors contributed equally to this work.

Abstract

Grassland is one of the most widely-distributed ecosystems on Earth and provides a variety of ecosystem services. Grasslands, however, currently suffer from severe degradation induced by human activities and climate change. In the present study, we explored the transcriptome response of *Stipa breviflora*, a dominant species in the desert steppe, to grazing through transcriptome sequencing, the development of simple sequence repeat (SSR) markers, and analysis of genetic diversity. *De novo* assembly produced 111,018 unigenes, of which 88,164 (79.41%) unigenes were annotated. A total of 686 unigenes showed significantly different expression under grazing, including 304 and 382 that were upregulated and downregulated, respectively. These differentially expressed genes (DEGs) were significantly enriched in the “alpha-linolenic acid metabolism” and “plant-pathogen interaction” pathways. Based on transcriptome sequencing data, we developed eight polymorphic molecular markers and investigated the genetic diversity of *S. breviflora* in grazed and ungrazed sites. We found that a relatively high level of *S. breviflora* genetic diversity occurred under grazing. This study enriches the knowledge of the genetic background of *S. breviflora*. The findings of genes that improve resistance to grazing are helpful for the restoration, conservation, and management of desert steppe.

Introduction

Chinese grasslands are diverse and include meadow grasslands, typical grasslands, desert grasslands, and alpine grasslands. The grasslands in China constitute the third largest grassland area worldwide, covering 41.7% of the country's territory and stretching from northern China to the Qinghai-Tibetan Plateau [1, 2]. These grasslands are currently in danger of degradation owing to climate change and anthropogenic activities, and over 33% of the degradation is due to overgrazing [3, 4]. Long-term overgrazing impacts species richness, community biomass, and soil quality [5-7]. However, plants can adapt to grazing pressure by developing certain characteristics, such as small sizes, fast growth, short life-spans, and low palatability, to avoid livestock feeding and increase the survival rate [8]. *Stipa* species are the most conspicuous grassland plants distributed throughout the Eurasian Steppe and are established as zonal vegetation in the Inner Mongolia grassland. *S. breviflora* Griseb. (Poaceae) is a dominant species and valuable forage resource in the desert steppe and is characterized by palatability, rich nutrient content, and early greening. Additionally, this species has strong ecological adaptability to diverse environments and can be a codominant species with *S. bungeana* and *S. krylovii* in warm-temperate steppe and typical steppe, respectively [9]. Although faced with pressures derived from grazing and climate change, this species has great potential for future

expansion [10]. Thus, there is a need for further elucidation of the adaptation of *S. breviflora* to grazing.

Genetic diversity is fundamental to biodiversity [11]. Climate factors, such as temperature and precipitation, and human activities impact genetic diversity, resulting in changes in genetic differentiation. Studies have shown that temperature is the key factor in determining the genetic differentiation of *S. breviflora* [9]. Anthropogenic activity, such as grazing, can increase, decrease, or have no significant effects on the genetic diversity of plants. In the *Stipa* genus, moderated grazing was found to promote the genetic diversity of *S. grandis* and *S. krylovii* [12]. Similar results were detected in the study by Shan et al. [13], and the genetic diversity of *S. grandis* under light grazing conditions was higher than that under no grazing. Other plants, such as *Elymus nutans*, had higher intrapopulation genetic diversity in three grazed areas than in ungrazed areas [14]. However, a negative relationship between grazing and genetic diversity was also observed. For example, *Artemisia frigida* displayed restricted gene flow and decreased genetic diversity under grazing [15]. In some cases, researchers found that grazing had no significant effect on plant genetic diversity. Smith et al. [16] investigated the fine-scale spatial genetic structure in perennial grasses and observed no differences in average gene diversity between populations of each species. A study on *Festuca idahoensis* revealed

that grazing did not significantly alter their genetic diversity [17]. In summary, although plants respond to climate and human activities by changing their genetic diversity, the molecular mechanisms driving these changes remain poorly understood.

With the popularity of next-generation sequencing (NGS), RNA-sequencing (RNA-seq) provides a new perspective for exploring the molecular mechanism of genetic diversity and microcosmic interactions in organisms. ~~This technique has been applied to plants such as *Arabidopsis* species [18], *Salvia miltiorrhiza* [19], *Zea mays* [20], and *Cajanus cajan* (L.) [21].~~ Using transcriptome sequencing, Ren et al. [22] developed 21 microsatellite markers of *S. breviflora* and tested the polymorphism in six related species. Transcriptome analysis has also been used to uncover the adaptive mechanisms of plants subjected to grazing. For *Leymus chinensis*, genes related to the systemic synthesis of jasmonate are activated during grazing [23]. Wang et al. [24] conducted *de novo* assembly to compare the transcriptomes of two alfalfa varieties that are tolerant and intolerant to grazing, and identified 21 differentially expressed responsive pathways. For *S. grandis*, individuals subjected to grazing grow significantly smaller than those not subjected to grazing, and the expression of wound-, drought-, and defense-related genes is altered [25]. However, the response of *S. breviflora* to grazing at the transcriptome level and the changes in genetic

diversity remain unclear.

Considering the crucial position of *S. breviflora* in desert steppe and to meet the needs for exploring the adaptation mechanism of grasses to grazing, we took this species as an example to (1) investigate the transcriptome response ~~of *S. breviflora*~~ to grazing; (2) develop simple sequence repeat (SSR) molecular markers based on transcriptome sequencing data; and (3) explore the impact of grazing on the genetic diversity ~~of *S. breviflora*~~. The present study provides insight into the adaptation mechanisms of plants under grazing.

Materials and methods

Collecting plant materials

The study area is in Wuchuan, Inner Mongolia Autonomous Region, China (41°47′-41°23′N, 110°31′-111°53′E) at an elevation of 1500-2000 m above sea level. This area belongs to the monsoon climate of medium latitudes. The mean annual precipitation and temperature are 354.1 mm and 2.6°C, respectively. We observed three ungrazed sites (codes 1, 2, and 3) and three grazed sites (codes 4, 5, and 6) (Table 1). The ungrazed sites have been fenced to prevent grazing for many years. In contrast, the grazed sites have been subjected to long-term heavy grazing. These sites are distributed adjacently and are approximately around 2-10 km apart, indicating that they are under similar backgrounds of temperature and precipitation.

Table 1. Information of the observed sites of *S. breviflora*.

Code	Position	Altitude(m)	Utilization	Plant community
1	111.21°E, 41.13°N	1629	ungrazing	*S. breviflora* + *S. krylovii*
2	111.26°E, 41.19°N	1681	ungrazing	*S. breviflora*, + *S. krylovii* + *Thymus mongolicus*
3	112.41°E, 41.17°N	1668	ungrazing	*S. breviflora* + *S. krylovii*
4	111.19°E, 41.11°N	1647	grazing	*S. breviflora*, + *S. krylovii* + *Thymus mongolicus*
5	111.29°E, 41.25°N	1614	grazing	*S. breviflora* + *Agropyron cristatum*
6	111.31°E, 41.29°N	1573	grazing	*S. breviflora*, + *S. krylovii* + *Cleistogenes squarrosa*

In June 2016, we collected 33 samples of leaves from healthy individuals at each site, with 3 samples for RNA-seq and 30 samples for genetic diversity analysis. Samples for RNA-seq were rinsed with RNase-free dd water, mixed with equal amounts, stored in dry ice, and sent to the Beijing Genomics Institute (BGI, Shenzhen, China) for total RNA extraction and high-throughput sequencing. When collecting other samples, we followed the principle of population genetics, choosing the individuals that were at least 10 m apart. All samples were immediately frozen in liquid nitrogen and brought back to the Genetics Lab and stored at -80 °C for genetic diversity analysis.

RNA preparation and cDNA library construction

The total RNA of each sample was extracted using Trizol reagent (Invitrogen, Carlsbad, CA, USA) according to the manufacturer's protocol. RNA integrity was examined using an Agilent 2100 Bioanalyzer (Agilent Technologies, Santa Clara, CA, USA), and poly (A) mRNA was isolated using Oligo (dT) beads. The mRNA fragments were used as templates for reverse transcription to obtain cDNA. After fragment ends were repaired, polymerase chain reaction (PCR) amplification was performed, followed by poly (A) ligation and fragment length selection to construct a cDNA library. To obtain a high-quality library, an Agilent 2100 Bioanalyzer and

ABI StepOnePlus real-time PCR system were used for quality control analysis.

RNA-seq and *de novo* assembly

The libraries were sequenced using the Illumina HiSeq 4000 platform (Illumina, USA). The raw reads generated by sequencing were filtered by eliminating adaptor sequences and low-quality reads and then assembled by the Trinity program with the default parameters [26]. The longest transcripts of each gene were chosen as unigenes.

Unigene annotation

Unigenes were aligned using the basic local search tool (BLAST) [27] against databases, including the nucleotide collection (Nt, https://www.ncbi.nlm.nih.gov/), National Center for Biotechnology Information (NCBI) non-redundant protein (Nr, https://www.ncbi.nlm.nih.gov/), Cluster of Orthologous Groups (COG, http://www.ncbi.nlm.nih.gov/COG), Kyoto Encyclopedia of Genes and Genomes (KEGG, http://www.genome.jp/kegg) and Swiss-Prot (http://ftp.ebi.ac.uk/pub/databases/swissprot) databases. Blast2GO [28] was used to annotate the unigenes against gene ontology (GO, http://geneontology.org) based on the results of the Nr annotation.

InterProScan5 [29] was used for InterPro (http://www.ebi.ac.uk/interpro) annotation. According to the functional annotation results, the best-

matched sequences were defined as complete coding sequences (CDS) of the corresponding unigenes and saved in FASTA format, with the following order of priority: Nr, SwissProt, KEGG, and COG. Moreover, the predicted CDS were used as a model to predict the unigenes without matches in the abovementioned database through modeling with ESTScan [30].

Differentially expressed gene (DEG) identification

The expression levels of each gene were calculated by RSEM [31]. Differential expression analysis of genes in grazed and ungrazed samples was performed using the NOIseq approach [32]. We set fold change ≥ 2.00 and probability ≥ 0.8 as thresholds to identify the genes that were expressed at a significantly different level.

Developing grazing-related SSRs

MISA [33] and Primer3 [34] were used to detect SSRs from the transcriptome and design primers, respectively. We selected 21 SSR molecular markers derived from DEGs to screen grazing-related SSRs and then determined the optimal annealing temperature by PCR amplification (S1 Table). Total genomic DNA was extracted using the TIANGEN plant genomic DNA kit (Tiangen Biotech, Beijing, China) for all samples following the manufacturer's instructions. The quality and quantity of DNA were determined by using a NanoDrop 2000 spectrophotometer (Thermo Scientific, Wilmington, DE, USA). The PCR amplification reaction mixture (25 μL)

contained 1 μL template DNA (30-40 ng μL–1), 0.5 μL (10 pM) of each primer, 12.5 μL Premix Taq (TaKaRa Biotechnology Co., Dalian, Liaoning Province, China), and 10.5 μL double distilled water (ddH2O). The PCR amplification parameters were as follows: 4 min at 94°C; 35 cycles of 30 s at 94°C, 30 s at a primer-specific annealing temperature, 30 s at 72°C and a final extension step at 72°C for 10 min. PCR products were then separated using capillary electrophoresis and genotyped using an ABI 3730 DNA analyzer with a GeneScan 500 LIZ size standard (Applied Biosystems, Beijing, China). Finally, we screened eight primers with polymorphic loci (Table 2). Polymorphism information content (PIC) values of markers were calculated using Cervus 3.0.7 [35].

Table 2. Characteristics of eight microsatellite loci for *S. breviflora*.

Gene ID	Primer sequence (5'→3')	Product size	Repeats	Ta(°C)[a]	PIC[b]
CL14453.Contig1	GAGGAAGCGTCGATCGTGAC	107	(CGG)7	61.5	0.47
	TGTCCACTTTCTGCTCCACG				
Unigene12747	GAGCGATGCAACGATTATATAGG	116	(CGACGC)5	58.3	0.38
	CATCGTGAAGTGATAAGAAGCCT				
CL616.Contig34	AATCAGCTCGTCGGTATTTGAT	124	(GT)6	53.0	0.33
	AGAGGGGAAGAACGAAATATCTG				
CL1837.Contig15	GGGGAGTTGGACTTGGTAGTG	150	(GGA)5	62.0	0.22
	CTTAACCTCCCTTCTCCACCTT				
CL15080.Contig2	ATCGTCAAACTCCACCTAATCAA	140	(TC)10	54.3	0.15
	AGGCAATATTGGCAACTCACTC				
CL966.Contig12	CACTGGGTTCTCTTCGTCTCC	237	(GA)7gg(GA)7	52.5	0.35

	TCTCCTCCTGCATCTTTCGTC				
CL.5285.Contig5	TATAGGCAGTGGGGAGACGA	192	(CTC)7	55.0	0.42
	AGCTTCGAGGGATGAGGAGA				
CL966.Contig15	CACCGGATGCAAAGAAACCG	221	(GA)7gg(GA)7	52.5	0.14
	CCTCCTGCATCTTTCGTCCTC				

[a] Annealing temperature.

[b] Polymorphism information content.

Genetic diversity analysis

We used the eight abovementioned polymorphic SSR markers to analyze genetic diversity among grazed and ungrazed samples. First, GeneMarker version 2.6.0 (SoftGenetics, State College, PA, USA) was used to perform peak identification and fragment sizing. Then, we applied GenAlEx 6.5 [36] to convert the data format and to calculate the genetic parameters, including the observed number of alleles (Na), effective number of alleles (Ne), expected heterozygosity (He) and observed heterozygosity (Ho), gene flow (Nm), and Shannon's information index (I).

Results

De novo assembly and unigene annotation

After sequence assembly and redundancy removal, we obtained 111,018 unigenes totaling 125,503,270 bp with an average length of 1130 bp. The N50 and CG contents were 1852 bp and 49.38%, respectively, which demonstrated adequate assembly quality (Table 3). For the functional annotation, 88,164 unigenes (79.41%) matched records in seven databases. The quantity of annotated unigenes in each database was as follows: Nr, 79,283 (71.41%); Nt, 83,097 (74.85%); SwissProt, 57,827 (52.09%); KEGG, 63,044 (56.79%); COG, 37,880 (34.12%); InterPro, 51,305 (46.21%); and GO, 50,089 (45.12%) (Table 4).

Table 3. Statistical summary of *S. breviflora* transcriptome data.

Assembly statistics	RNA-seq
Number of unigenes	111,018
Total length (bp)	125,503,270
Mean length (bp) [a]	1130
N50 (bp)[b]	1852
GC (%)[c]	49.38

[a] Mean length of the assembled sequences.

[b] The length of the content or unigene corresponding to the sequence, which is added to 50% of the total assembled bases when the assembled sequences are sorted from short to long.

[c] GC content.

Table 4. Summary of functional annotations of the assembled unigenes.

Database	Number of unigene hits	Percentage (%)
Nr	79,283	71.41
Nt	83,097	74.85
SwissProt	57,827	52.09
KEGG	63,044	56.79
COG	37,880	34.12
Interpro	51,305	46.21
GO	50,089	45.12
Overall	88,164	79.41

Based on the NR annotation, we found species with genes homologous to *S. breviflora*. The results showed that 46.02% of unigene sequences matched *Brachypodium distachyon*, followed by other species (28.93%), *Hordeum vulgare subsp. Vulgare* (11.06%), *Oryza sativa Japonica* group (7.15%), and *Aegilops tauschii* (6.84%) (Fig 1).

Fig 1. Unigene homology searches against the NR database.

We matched unigenes to the GO database to clarify the cellular function of the gene products. We found that 50,089 unigenes were matched to

more than 50 functional items, which were divided into three main groups: biological process, cellular component, and molecular function (Fig 2). The largest subcategory of biological process was “metabolic process” (26,800), followed by “cellular process” (26,513). Of the cellular component, “cell” and “cell parts” showed the most matches with unigenes at 26,939 and 26,833, respectively. Among the molecular function category, “binding” and “catalytic activity” showed the most matches at 24,708 and 22,732, respectively.

Fig 2. GO classifications of the assembled unigenes.

With the aim of validating protein functions, the COG database was used to compare with unigenes. A total of 37,880 unigenes were annotated into 25 COG functional categories (Fig 3). The “general function prediction only” was the cluster with the most unigenes (10,786), followed by “transcription” (7184), “translation, ribosomal structure and biogenesis” (7048), “cell cycle control” (6488), and “function unknown” (6314), whereas only a few unigenes were assigned to “extracellular structures” and “nuclear structure”.

Fig 3. COG classifications of the assembled unigenes.

KEGG annotation was performed to identify the metabolic pathway to which unigenes belonged, and 63,044 unigenes had matches in the KEGG database. These unigenes were assigned to 133 pathways.

Differential expression and pathway analysis

Using the NOI approach, we identified 686 DEGs, including upregulated (304 DEGs) and downregulated (382 DEGs). Then, a similarity search of the DEGs in the KEGG database was performed to explore the pathways associated with a significant enrichment in response to grazing. We found that these DEGs were sorted into 100 pathways. As presented in Table 5, the main enriched pathways included “alpha-linolenic acid metabolism” and “plant-pathogen interaction”.

Table 5. The top ten KEGG enriched pathway of DEGs.

KEGG terms	Gene number	Q value	Pathway ID
Alpha-linolenic acid metabolism	13	2.90E-05***	ko00592
Plant-pathogen interaction	57	9.96E-04***	ko04626
Pentose and glucuronate interconversions	13	0.12	ko00040
Diterpenoid biosynthesis	5	0.12	ko00904
Biosynthesis of secondary metabolites	80	0.18	ko01110
Starch and sucrose metabolism	20	0.23	ko00500
Linoleic acid metabolism	4	0.27	ko00591
Phenylpropanoid biosynthesis	25	0.61	ko00940
Sesquiterpenoid and triterpenoid biosynthesis	3	0.61	ko00909
Glutathione metabolism	7	0.61	ko00480

***q<0.001, significantly enriched pathway of DEGs.

Development and validation of grazing-related SSR markers

MISA was used to mine SSR markers from transcriptomes using the following criteria: mono-nucleotides with at least 12 repeats, di-nucleotides with 6 repeats, tri- and tetra-nucleotides with 5 repeats, and penta- and hexa-nucleotides with 4 repeats. We examined 111,018 sequences and identified 19,607 SSRs (S2 Table). Additionally, 11,963 tri-nucleotide SSRs and 386 quad-nucleotide SSRs accounted for 61.01% and 1.97% of all the SSRs, respectively, representing the most and the least of all SSR markers. The CCG/CGG trinucleotide repeats were the most abundant motifs detected in SSRs (5186, 26.45%), followed by the AG/CT (3117, 15.90%), AGG/CCT (2611, 13.32%), and AGC/CTG (1716, 8.75%) motifs (Table 6). The frequency of the remaining motifs accounted for 35.58% of the SSRs.

Table 6. Distribution of simple sequence repeat types in *S. breviflora* transcriptome.

SSR motif	SSR motif numbers	Ratio (%)
Mono-nucleotide	1059	5.40
AC/GT	678	3.46
AG/CT	3117	15.90
AT/AT	624	3.18
CG/CG	377	1.92

Tri-nucleotide	11,963	61.01
AAC/GTT	149	0.76
AAG/CTT	576	2.94
AAT/ATT	52	0.27
ACC/GGT	812	4.14
ACG/CGT	545	2.78
ACT/AGT	52	0.27
AGC/CTG	1716	8.75
AGG/CCT	2611	13.32
ATC/ATG	264	1.35
CCG/CGG	5186	26.45
Quad–nucleotide	386	1.97
Penta–nucleotide	542	2.76
Hexa–nucleotide	861	4.39

To develop grazing-related molecular markers, we preliminarily selected 21 DEGs and designed primers (S1 Table). After screening polymorphic SSRs, excluding primers with product sizes that did not meet the requirements and could not generate PCR products, eight grazing-related genes with SSR polymorphisms were found. In this study, PIC values ranged from 0.14 to 0.47, averaging 0.31, which indicated that these markers were informative and effective for genetic analysis (Table 2).

Effects of grazing on genetic diversity

The eight primers yielded 29 alleles for 180 samples from the six sites. Among these primers, CL966.Contig12 and CL.5285.Contig5 produced the most alleles with 5. The fewest numbers of alleles were exhibited in CL1837.Contig15 and Unigene12747 with 2 (Table 7). On average, there were 4 alleles per primer, suggesting lower polymorphism than the results reported by Ren et al. [22], who reported 6 alleles per primer. Shannon's information index of the primers ranged from 0.31 to 0.92, with a mean of 0.62.

Table 7. Diversity statistics of the eight SSR markers across *S. breviflora* populations.

Locus	Na[a]	Ne[b]	Ho[c]	He[d]	I[e]	Nm[f]
CL14453.Contig1	4	2.23	0.05	0.55	0.92	6.76
Unigene12747	2	2.00	0.02	0.50	0.69	4.28
CL616.Contig34	4	1.63	0.02	0.39	0.67	4.16
CL1837.Contig15	2	1.33	0.00	0.25	0.41	11.57
CL15080.Contig2	3	1.19	0.05	0.16	0.34	14.99
CL966.Contig12	5	1.64	0.00	0.39	0.73	11.52
CL.5285.Contig5	5	1.90	0.13	0.47	0.88	6.26
CL966.Contig15	4	1.17	0.00	0.14	0.31	7.11
mean	4	1.64	0.03	0.36	0.62	8.33

[a] Observed number of alleles.

[b] Effective number of alleles.

[c] Observed heterozygosity.

[d] Expected heterozygosity.

[e] Shannon's information index.

[f] Gene flow.

The genetic diversity of *S. breviflora* can be calculated using Shannon's information index (I) for each population. As Table 8 indicates, the grazed populations showed the highest Shannon's information index value, with a value of 0.64 (pop4 and pop6). In contrast, the ungrazed populations showed the lowest level of genetic diversity, with a value of 0.44 (pop2). The observed numbers of alleles (Na) ranged from 2.13 (pop2) to 3.00 (pop4), with an average of 2.71. The effective number of alleles (Ne) varied from 1.47 (pop2) to 1.71 (pop4), with an average of 1.62. Compared with the mean observed heterozygosity of 0.04 (Ho), the mean expected heterozygosity (He) value was high, with a value of 0.34. This result indicated that the inbreeding rate of *S. breviflora* is high, and the proportion of heterozygotes is relatively low. The results showed that grazed populations had higher genetic diversity than ungrazed populations.

Table 8. Genetic diversity statistics of *S. breviflora* populations.

Code	Population size	Na[a]	Ne[b]	Ho[c]	He[d]	I[e]	PPB (%)[f]
1	29	2.50	1.63	0.05	0.32	0.55	100.00
2	29	2.13	1.47	0.02	0.28	0.44	87.50
3	27	2.88	1.61	0.05	0.33	0.51	100.00
4	30	3.00	1.71	0.0[illegible]	0.38	0.64	100.00
5	28	2.88	1.61	0.03	0.36	0.62	100.00
6	28	2.88	1.66	0.03	0.38	0.64	100.00
mean	29	2.71	1.62	0.04	0.34	0.57	97.92

[a]Na: observed number of alleles.

[b]Ne: effective number of alleles.

[c]Ho: observed heterozygosity.

[d]He: expected heterozygosity.

[e]I: Shannon's information index.

[f]PPB: percentage of polymorphic bands.

Discussion

NGS strategies and their application to transcriptomics can be used to meet the increasing demands of acquiring accurate genome information. To clarify the transcriptome responses of *S. breviflora* to grazing, we compared the transcriptome changes under grazing and nongrazing conditions and found that 686 unigenes showed differential expression. These DEGs

were significantly enriched in the “alpha-linolenic acid and metabolism” and “plant-pathogen interaction” pathways, which may enhance the adaptability of *S. breviflora* to grazing. Based on DEGs, we screened eight grazing-related SSR markers to explore the genetic diversity of *S. breviflora* and found that grazing has a positive effect on genetic diversity. Changes in gene expression levels help *S. breviflora* defend against grazing damage, and the increased genetic diversity provides the potential for its adaptation and evolution; all of these factors contribute to the survival of plants in different aspects.

Grazing stimulates the expression of wound and defense-related genes

Wounding is an inevitable threat that occurs over the entire life-span of plants, especially under grazing conditions [25]. In addition to developing adaptive traits such as low heights, small leaf sizes, low root biomass [37], few vegetative tillers, and short internode lengths [38], plants also modulate the expression levels of related genes, for instance, genes related to the jasmonic acid (JA) family and plant immunity [39], to defend themselves against grazing damage. JA is a typical wound hormone derived from linolenic acid, and its accumulation could modulate the expression of wounding-response genes [40]. JA also plays an important role in coordi-

nating growth and immune responses to increase plant survival in unpredictable environments [41]. In *acx1/5* double mutants, JA deficiency decreases resistance to leaf-eating insects. Accumulation of *acx1/5* in plants can induce responses to infections by a fungal pathogen [42]. Genes such as lipoxygenase (*LOX*), fatty acid desaturases (*FAD*), and allene oxide synthase (*AOS*), which are known to be involved in the synthesis or metabolism of members of the JA family, are also induced when the leaves of *Arabidopsis* are wounded [43]. *PLA1* and *13-LOX* belong to the "alpha-linolenic acid metabolism" pathway, and *MAPKK* is a key gene in the "plant-pathogen interaction" pathway. In the present study, we observe[illegible]hat these genes were upregulated, which is consistent with the abovementioned observations.

PAL1 acts as a digestive enzyme that hydrolyzes phospholipids and produces 2-acyl-lysophospholipids and fatty acids (FAs). PAL1 exists in nearly all cell types and is involved in membrane maintenance, remodeling, and the regulation of cellular mechanism [44]. In plants, the PAL1 reaction produces FA products for further regulation of JA biosynthesis [44]. Singh et al. [45] elucidated the expression profile of rice PLA-encoding genes under salt, cold, and drought stress using microarray data and found that all the DEGs were upregulated. *LOXs* function as mediators that catalyze the oxygenation of FA and promote the production of oxylipins such as JA

[46]. As a primary family member of *LOX*, *13-LOX* uses 13(S)-hydroperoxy-octadecatrienoic acid to catalyze the biosynthesis of JA [47]. In maize, the ZmLOX10 protein has been suggested to be a linoleate 13-LOX. When maize is subjected to mechanical damage and cold stress, *ZmLOX10* is preferentially expressed [48]. When tomato (*Solanum lycopersicum L.*) is exposed to methyl-jasmonate treatment and damaged, *SILOX4*, which belongs to the *13-LOX* subfamily, is highly expressed [46]. In other plants, such as rice [49], citrus [50], and cotton [46], increased *LOX* activity is also observed, which is considered to be directly correlated with plant tolerance to biotic and abiotic stress.

Plants are injured by defoliation when animal graze. This injury provides opportunities for pathogens to infect plants. External microorganisms in feces and urine increase the risk of invasion by pathogens [51]. When plants are infected by pathogens, they develop disease resistance by activating mitogen-activated protein kinase (MAPK) cascades [52]. Specifically, MAPK kinase kinases (MAPKKKs) phosphorylate MAPK kinases (MAPKKs), which then phosphorylate MAPKs [53]. In the present study, *MAPKK* genes showed upregulated expression under grazing, suggesting that *MAPKK* genes contribute to the defense against invasion by microorganisms. Gene overexpression in the MAPK cascade defense mechanism occurs widely in plants, such as rice [54], *Arabidopsis* [55], cotton [56],

and wheat [57].

Overall, altering the key gene expression levels in response to biotic and abiotic stress is a survival strategy for plants. When *S. breviflora* is exposed to grazing str[illegible] it adopts this strategy to activate relevant genes, such as *PLA1*, *13-LOX*, and *MAPKK*, to stay alive.

Grazing increases the genetic diversity of *S. breviflora*

Genetic diversity is a fundamental basis for plants to adapt to the environment, and to provide the potential for evolution. Genetic diversity is shaped by the balance between genetic drift, inbreeding, recombination, gene flow, mutation, and selection [58]. Mammal grazing is a complex process that can be divided into three parts: foliar tissue removal (defoliation), nutrient return via dung and urine, and trampling [51]. Therefore, grazing, as a disturbance, affects plant genetic diversity in different ways. Basically, genome-wide selection occurs during grazing. The substitution rate of mutants increases in the process of livestock feeding [59]. On the other hand, when accompanied by trampling and livestock migration, population genetic diversity changes due to gene flow caused by seed dispersal.

During grazing, defoliation and trampling cause direct mechanical damage to the plant, consequently creating more chances for the microorganisms in the environment to invade plants through open wounds. In response to grazing, *S. breviflora* not only develops resistance capabilities by

regulating gene expression but also exhibits changes in DNA sequences. This conclusion can be attributed to the fact that the SSR markers used in this study were mined from DEGs. As a result, the polymorphisms of these SSR markers, to some extent, also reflect that the DNA sequences of grazing-related genes have changed to resist grazing pressure. This finding suggests that grazing acts as the selection process that can shape the genotypes of *S. breviflora*.

Gene flow is an indispensable force that influences genetic diversity of the natural population. Generally, high gene flow leads to high genetic diversity. This phenomenon is supported in many plants, such as *E. nutans* [14] and *S. purpurea* [60]. Gene flow occurs through different media, such as pollen, seeds, spores, and other genetic materials. Because *S. breviflora* is a self-pollinating species, seed diffusion is the major method of genetic material transmission. During grazing, livestock promotes seed dispersal, thereby enhancing seed-mediated gene flow and eventually leading to increased genetic diversity. As a parameter to quantify gene flow, Nm is considered the number of migrants per generation. When Nm is more than one, gene flow could counteract the effects of genetic drift and thus prevent genetic differentiation. In contrast, if Nm is less than one, genetic drift could lead to genetic differentiation among populations. In this study, we observed a much higher gene flow between primers in *S. breviflora* than that

in other *Stipa* plants, such as *S. grandis* [13] and *S. krylovii* [12], which varied from 4.16 to 14.99, with a mean value of 8.33 (Table 7). This finding is probably because the SSR system is more efficient than the ISSR system in accessing genetic analysis [61]. From the standpoint of gene flow, grazing is an important way to maintain and conserve the genetic diversity of *S. breviflora*. Additionally, livestock trampling blocks the clonal growth of plants and ultimately cause an increase in genetic diversity by sexual reproduction [62].

The aim of the present study was to evaluate the effects of grazing on genetic diversity. The grazing intensity was not considered in our study. However, grazing intensity has been taken into account in other studies. Shan et al. [13] indicated that the genetic diversity of *S. grandis* was the highest under light grazing. Similar results were suggested for *E. nutans* in the study by Ma et al. [14]. For *S. krylovii* [12] and *S. purpurea* [60], the highest value appeared in moderately grazed populations. Moreover, contradictory results, including a negative relationship [63] and no correlation [64], were reported in other studies. These contradictory results reflect the complex relationship between grazing and plant genetic diversity, which are affected by grazing intensity, differences in species, geographic locations and molecular marker systems. From the facts above, the protection

of desert steppe requires a deeper understanding of the genetic characteristics of *S. breviflora*, and the implementation of sustainable management in conjunction with the local environment.

Conclusions

Overall, grazing activated plant defense-related genes and increased genetic diversity in *S. breviflora*. The desert steppe in Inner Mongolia has become severely degraded because of long-term overgrazing. The *S. breviflora* community, however, remains stable, and this species codominates with *S. bungeana*, *S. klemenzii*, and *S. krylovii* in different types of steppe under diverse conditions of precipitation, temperature, and soil attributes. The reason for this may be explained in part by our findings, i.e., the enhancement of genetic diversity of *S. breviflora* under grazing improves the adaptability of the species to stressed environments. This study not only enriches the genetic background for *S. breviflora* but is also helpful for the restoration, conservation, and management of desert steppe.

References

1. Fang J, Yang Y, Ma W, Mohammat A, Shen H. Ecosystem carbon stocks and their changes in China's grasslands. Science China Life Science. 2010;53(7):757–65. https://dio.org/10.1007/s11427-010-4029-x PMID:20697865.
2. Yang Y, Fang J, Ma W, Smith P, Mohammat A, Wang S, et al. Soil carbon stock and its

changes in northern China's grasslands from 1980s to 2000s. Global Change Biol. 2010;16(11):3036–47. https://dio.org/10.1111/j.1365-2486.2009.02123.x.
3. Akiyama T, Kawamura K. Grassland degradation in China: Methods of monitoring, management and restoration. Grassland Science. 2007;53(1):1–17. https://dio.org/10.1111/j.1744-697X.2007.00073.x.
4. Guo Z, Liang T, Zhang Z. Classification management for grassland in Gansu Province, China. N Z J Agric Res. 2010;46(2):123–31. https://dio.org/10.1080/00288233.2003.9513538.
5. Bai Y, Wu J, Clark CM, Pan Q, Zhang L, Chen S, et al. Grazing alters ecosystem functioning and C:N:P stoichiometry of grasslands along a regional precipitation gradient. J Appl Ecol. 2012;49(6):1204–15. https://dio.org/10.1111/j.1365-2664.2012.02205.x.
6. Zhang B, Thomas BW, Beck R, Liu K, Zhao M, Hao X. Labile soil organic matter in response to long–term cattle grazing on sloped rough fescue grassland in the foothills of the Rocky Mountains, Alberta. Geoderma. 2018;318:9–15. https://dio.org/10.1016/j.geoderma.2017.12.019.
7. Zhang T, Zhao H, Li S, Zhou R. Grassland changes under grazing stress in Horqin sandy grassland in Inner Mongolia, China. N Z J Agric Res. 2010;47(3):307–12. https://dio.org/10.1080/00288233.2004.9513599.
8. Evju M, Austrheim G, Halvorsen R, MysterudAtle M. Grazing responses in herbs in relation to herbivore selectivity and plant traits in an alpine ecosystem. Oecologia. 2009;161(1):77–85. https://dio.org/10.1007/s00442-009-1358-1.
9. Zhang Q, Niu JM, Wu SB, Buyantuyev A, Dong JJ. Impact of climatic factors on genetic diversity of *Stipa breviflora* populations in Inner Mongolia. Gen Mol Res. 2012;11(3):2081–93. https://dio.org/10.4238/2012.August.6.12 PMID:22911592.
10. Lv X, Zhou G. Climatic Suitability of the Geographic Distribution of *Stipa breviflora* in Chinese Temperate Grassland under Climate Change. Sustainability. 2018;10(10):3767. https://dio.org/10.3390/su10103767.
11. Pauls SU, Nowak C, Balint M, Pfenninger M. The impact of global climate change on genetic diversity within populations and species. Mol Ecol. 2013;22(4):925–46. https://dio.org/10.1111/mec.12152 PMID:23279006.
12. Peng J, Liang C, Niu Y, Jiang W, Wang W, Wang L. Moderate grazing promotes genetic diversity of *Stipa* species in the Inner Mongolian steppe. Landscape Ecol. 2015;30(9):1783–94. https://dio.org/10.1007/s10980-015-0227-z.
13. Shan D, Zhao M, Han B, Han G. Examining the genetic diversity of *Stipa grandis* under various grazing pressures. Acta Ecologica Sinica. 2006;26(10):3175–82. https://dio.org/10.1016/s1872-2032(06)60048-6.
14. Ma D, Guo Y, Hou F, Zhai X, Wang W, Tian M, et al. Plant genetic diversity and grazing management on the Qinghai–Tibetan Plateau: A case study of a dominant native wheatgrass (*Elymus nutans*). Biochem Syst Ecol. 2014;56:16–23. https://dio.org/10.1016/j.bse.2014.04.014.
15. Wang J, Yang C, Yin J, Wang TJ, Liu PT. Changes of the genetic diversity of Aremisia frigida population under the disturbance of grazing. Acta Ecologica Sinica. 2004;24(11):2465–71.
16. Smith SE, Arredondo T, Aguiar M, Huber-Sannwald E, Alpuche A, Aguado A, et al. Fine-

Scale Spatial Genetic Structure in Perennial Grasses in Three Environments. Rangeland Ecol Manage. 2009;62(4):356-63. https://dio.org/10.2111/08-159.1.

17. Matlaga D, Karoly K. Long–term grazing effects on genetic variation in Idaho fescue. Journal of Range Management. 2004;57(3):275–9. https://dio.org/10.2111/1551-5028(2004)057[0275:lgeogv]2.0.co;2.
18. Rasmussen S, Barah P, Suarez–Rodriguez MC, Bressendorff S, Friis P, Costantino P, et al. Transcriptome Responses to Combinations of Stresses in *Arabidopsis*. Plant Physiol. 2013;161(4):1783–94. https://dio.org/10.1104/pp.112.210773.
19. Xu Z, Peters RJ, Weirather J, Luo H, Liao B, Zhang X, et al. Full–length transcriptome sequences and splice variants obtained by a combination of sequencing platforms applied to different root tissues of *Salvia miltiorrhiza* and tanshinone biosynthesis. The Plant Journal. 2015;82(6):951–61. https://dio.org/10.1111/tpj.12865.
20. Stelpflug SC, Sekhon RS, Vaillancourt B, Hirsch CN, Buell CR, Leon ND, et al. An Expanded Maize Gene Expression Atlas based on RNA Sequencing and its Use to Explore Root Development. The Plant Genome. 2016;9(1):16. https://dio.org/10.3835/plantgenome2015.04.0025.
21. Dutta S, Kumawat G, Singh BP, Gupta DK, Singh S, Dogra V, et al. Development of genic–SSR markers by deep transcriptome sequencing in pigeonpea [*Cajanus cajan* (L.) Millspaugh]. BMC Plant Biol. 2011;11(1):17. https://dio.org/10.1186/1471-2229-11-17.
22. Ren J, Su Z, Dang z, Ding y, Wang P, Niu J. Development and characterization of EST–SSR markers in *Stipa breviflora* (Poaceae). Applications in Plant Sciences. 2017;5(4):1600157. https://dio.org/10.3732/apps.1600157 PMID:28439477.
23. Chen S, Cai Y, Zhang L, Yan X, Cheng L, Qi D, et al. Transcriptome analysis reveals common and distinct mechanisms for sheepgrass (*Leymus chinensis*) responses to defoliation compared to mechanical wounding. PLoS One. 2014;9(2):e89495. https://dio.org/10.1371/journal.pone.0089495.
24. Wang J, Zhao Y, Ray I, Song M. Transcriptome responses in alfalfa associated with tolerance to intensive animal grazing. Scientific Reports. 2016;6:19438. https://dio.org/10.1038/srep19438 PMID:26763747.
25. Wan D, Wan Y, Hou X, Ren W, Ding Y, Sa R. *De novo* assembly and transcriptomic profiling of the grazing response in *Stipa grandis*. PLoS One. 2015;10(4):e0122641. https://dio.org/10.1371/journal.pone.0122641.
26. Grabherr MG, Haas BJ, Yassour M, Levin JZ, Thompson DA, Amit I, et al. Full–length transcriptome assembly from RNA–Seq data without a reference genome. Nat Biotechnol. 2011;29:644–54. https://dio.org/10.1038/nbt.1883.
27. Altschul SF, Gish W, Miller W, Myers EW, Lipman DJ. Basic local alignment search tool. J Mol Biol. 1990;215(3):403–10. https://dio.org/10.1016/S0022-2836(05)80360-2.
28. Conesa A, Gotz S, Garcia–Gomez JM, Terol J, Talon M, Robles M. Blast2GO: a universal tool for annotation, visualization and analysis in functional genomics research. Bioinformatics. 2005;21(18):3674–6. https://dio.org/10.1093/bioinformatics/bti610 PMID:16081474.
29. Quevillon E, Silventoinen V, Pillai S, Harte N, Mulder N, Apweiler R, et al. InterProScan: protein domains identifier. Nucleic Acids Res. 2005;33(Web Server issue):116–20. https://dio.org/10.1093/nar/gki442 PMID:15980438.

30. Iseli C, Jongeneel CV, Bucher P. ESTScan: a program for detecting, evaluating, and reconstructing potential coding regions in EST sequences. Proceedings International Conference on Intelligent Systems for Molecular Biology. 1999:138–48. PMID:10786296.
31. Li B, Dewey CN. RSEM: accurate transcript quantification from RNA–Seq data with or without a reference genome. BMC Bioinformatics. 2011;12:323–. https://dio.org/10.1186/1471-2105-12-323 PMID:21816040.
32. Tarazona S, Garcia–Alcalde F, Dopazo J, Ferrer A, Conesa A. Differential expression in RNA–seq: a matter of depth. Genome Res. 2011;21(12):2213–23. https://dio.org/10.1101/gr.124321.111.
33. Thiel T, Michalek W, Varshney R, Graner A. Exploiting EST databases for the development and characterization of gene–derived SSR–markers in barley (*Hordeum vulgare* L.). Theor Appl Genet. 2003;106(3):411–22. https://dio.org/10.1007/s00122-002-1031-0.
34. Untergasser A, Cutcutache I, Koressaar T, Ye J, Faircloth BC, Remm M, et al. Primer3–new capabilities and interfaces. Nucleic Acids Res. 2012;40(15):e115. https://dio.org/10.1093/nar/gks596.
35. Kalinowski ST, Taper ML, Marshall TC. Revising how the computer program CERVUS accommodates genotyping error increases success in paternity assignment. Mol Ecol. 2007;16(5):1099-106. https://dio.org/10.1111/j.1365-294X.2007.03089.x PMID:17305863.
36. Peakall R, Smouse PE. GenAlEx 6.5: genetic analysis in Excel. Population genetic software for teaching and research–an update. Bioinformatics. 2012;28(19):2537–9. https://dio.org/10.1093/bioinformatics/bts460 PMID:22820204.
37. Zuo X, Zhang J, Lv P, Wang S, Yang Y, Yue X, et al. Effects of plant functional diversity induced by grazing and soil properties on above–and belowground biomass in a semiarid grassland. Ecol Indicators. 2018;93:555–61. https://dio.org/10.1016/j.ecolind.2018.05.032.
38. Zhao W, Chen SP, Han XG, Lin GH. Effects of long–term grazing on the morphological and functional traits of *Leymus chinensis* in the semiarid grassland of Inner Mongolia, China. Ecol Res. 2008;24(1):99–108. https://dio.org/10.1007/s11284-008-0486-0.
39. Tsuda K, Katagiri F. Comparing signaling mechanisms engaged in pattern–triggered and effector–triggered immunity. Curr Opin Plant Biol. 2010;13(4):459–65. https://dio.org/10.1016/j.pbi.2010.04.006.
40. Cheong YH, Chang HS, Gupta R, Wang X, Zhu T, Luan S. Transcriptional profiling reveals novel interactions between wounding, pathogen, abiotic stress, and hormonal responses in *Arabidopsis*. Plant Physiol. 2002;129(2):661–77. https://dio.org/10.1104/pp.002857 PMID:12068110.
41. Howe GA, Major IT, Koo AJ. Modularity in Jasmonate Signaling for Multistress Resilience. In: Merchant SS, editor. Annual Review of Plant Biology, Vol 69. Annual Review of Plant Biology. 69. Palo Alto: Annual Reviews; 2018. p. 387–415.
42. Schilmiller AL, Koo AJK, Howe GA. Functional diversification of acyl–coenzyme A oxidases in jasmonic acid biosynthesis and action. Plant Physiol. 2007;143(2):812–24. https://dio.org/10.1104/pp.106.092916 PMID:17172287.
43. Reymond P, ., Weber H, ., Damond M, ., Farmer EE. Differential gene expression in response to mechanical wounding and insect feeding in *Arabidopsis*. The Plant Cell. 2000;12(5):707–19. https://dio.org/10.1105/tpc.12.5.707.
44. Richmond GS, Smith TK. Phospholipases A_1. International Journal of Molecular Sciences.

2011;12(1):588–612. https://dio.org/10.3390/ijms12010588.

45. Singh A, Baranwal V, Shankar A, Kanwar P, Ranjan R, Yadav S, et al. Rice phospholipase A superfamily: organization, phylogenetic and expression analysis during abiotic stresses and development. PLoS One. 2012;7(2):e30947. https://dio.org/10.1371/journal.pone.0030947 PMID:22363522.
46. Upadhyay RK, Mattoo AK. Genome–wide identification of tomato (*Solanum lycopersicum* L.) lipoxygenases coupled with expression profiles during plant development and in response to methyl–jasmonate and wounding. J Plant Physiol. 2018;231:318–28. https://dio.org/10.1016/j.jplph.2018.10.001.
47. Schaller F. Enzymes of the biosynthesis of octadecanoid–derived signalling molecules. Journal of Experimental Botany. 2001;52(354):11–23. https://dio.org/10.1093/jexbot/52.354.11.
48. Nemchenko A, Kunze S, Feussner I, Kolomiets M. Duplicate maize 13–lipoxygenase genes are differentially regulated by circadian rhythm, cold stress, wounding, pathogen infection, and hormonal treatments. J Exp Bot. 2006;57(14):3767–79. https://dio.org/10.1093/jxb/erl137 PMID:17005920.
49. Mostofa MG, Hossain MA, Fujita M. Trehalose pretreatment induces salt tolerance in rice (*Oryza sativa* L.) seedlings: oxidative damage and co–induction of antioxidant defense and glyoxalase systems. Protoplasma. 2015;252(2):461-75. https://dio.org/10.1007/s00709-014-0691-3 PMID:25164029.
50. Ben–Hayyim G, Gueta–Dahan Y, Avsian–Kretchmer O, Weichert H, Feussner I. Preferential induction of a 9–lipoxygenase by salt in salt–tolerant cells of *Citrus sinensis* L. Osbeck. Planta. 2001;212(3):367–75. https://dio.org/10.1007/s004250000397.
51. Sørensen LI, Mikola J, Kytöviita MM, Olofsson J. Trampling and Spatial Heterogeneity Explain Decomposer Abundances in a Sub–Arctic Grassland Subjected to Simulated Reindeer Grazing. Ecosystems. 2009;12(5):830–42. https://dio.org/10.1007/s10021-009-9260-6.
52. Bi G, Zhou Z, Wang W, Li L, Rao S, Wu Y, et al. Receptor–Like Cytoplasmic Kinases Directly Link Diverse Pattern Recognition Receptors to the Activation of Mitogen–Activated Protein Kinase Cascades in *Arabidopsis*. The Plant Cell. 2018;30(7):1543–61. https://dio.org/10.1105/tpc.17.00981 PMID:29871986.
53. Hashimoto M, Komatsu K, Maejima K, Okano Y, Shiraishi T, Ishikawa K, et al. Identification of three MAPKKKs forming a linear signaling pathway leading to programmed cell death in *Nicotiana benthamiana*. BMC Plant Biol. 2012;12:13. https://dio.org/10.1186/1471-2229-12-103.
54. Xiong LZ, Yang YN. Disease resistance and abiotic stress tolerance in rice are inversely modulated by an abscisic acid–inducible mitogen–activated protein kinase. The Plant Cell. 2003;15(3):745–59. https://dio.org/10.1105/tpc.008714.
55. Ren D, Liu Y, Yang KY, Han L, Mao G, Glazebrook J, et al. A fungal–responsive MAPK cascade regulates phytoalexin biosynthesis in *Arabidopsis*. Proceedings of the National Academy of Sciences of the United States of America. 2008;105(14):5638–43. https://dio.org/10.1073/pnas.0711301105 PMID:18378893.
56. Meng J, Gao H, Zhai WB, Shi JY, Zhang MZ, Zhang WW, et al. Subtle regulation of cotton resistance to Verticillium wilt mediated by MAPKK family members. Plant Sci.

2018;272:235–42. https://dio.org/10.1016/j.plantsci.2018.05.003 PMID:WOS:000437372800025.
57. Zhu X, Qi T, Yang Q, He F, Tan C, Ma W, et al. Host–induced gene silencing of the MAPKK gene *PsFUZ7* confers stable resistance to wheat stripe rust. Plant Physiol. 2017;175(4):1853–63. https://dio.org/10.1104/pp.17.01223 PMID:29070517.
58. Loveless MD, Hamrick JL. Ecological Determinants of Genetic Structure in Plant Populations. Annual Review of Ecology, Evolution, and Systematics. 1984;15(1):65–95.
59. Marcotrigiano M, . Herbivory could unlock mutations sequestered in stratified shoot apices of genetic mosaics. Am J Bot. 2000;87(3):355–61.
60. Zhai X-Y, Guo Y-X, Hou F-J, Liu Y, Ma D-T, Wang W, et al. Population genetic structure and germplasm conservation of Stipa purpurea on Qinghai-Tibetan Plateau under grazing. Biochem Syst Ecol. 2015;62:51-7. https://dio.org/10.1016/j.bse.2015.07.036.
61. Wang JS, He JH, Chen HR, Chen YY, Qiao F. Genetic Diversity in Various Accessions of Pineapple [Ananas comosus (L.) Merr.] Using ISSR and SSR Markers. Biochem Genet. 2017;55(5-6):347-66. https://dio.org/10.1007/s10528-017-9803-z PMID:28478603.
62. Jing Z, Yu J, Cheng J. Genetic diversity of a dominant species *Stipa bungeana* and its conservation strategy in the Loess Plateau of China. Biochem Syst Ecol. 2013;47:126–31. https://dio.org/10.1016/j.bse.2012.10.004.
63. Souto CP, Tadey M. Livestock Effects on Genetic Variation of Creosote Bushes in Patagonian Rangelands. Environ Conserv. 2018;46(1):59–66. https://dio.org/10.1017/s0376892918000280.
64. Jamnadass R, Mace ES, Hiernaux P, Muchugi A, Hanson J. Population genetic responses of wild forage species to grazing along a rainfall gradient in the Sahel: A study combining phenotypic and molecular analyses. Euphytica. 2006;151(3):431–45. https://dio.org/10.1007/s10681-006-9175-7.

Supporting information

S1 Table. Twenty-one candidate microsatellite loci for *S. breviflora.*

S2 Table. Frequency of classified repeat types.

 Click here to download Figure Fig1.tif

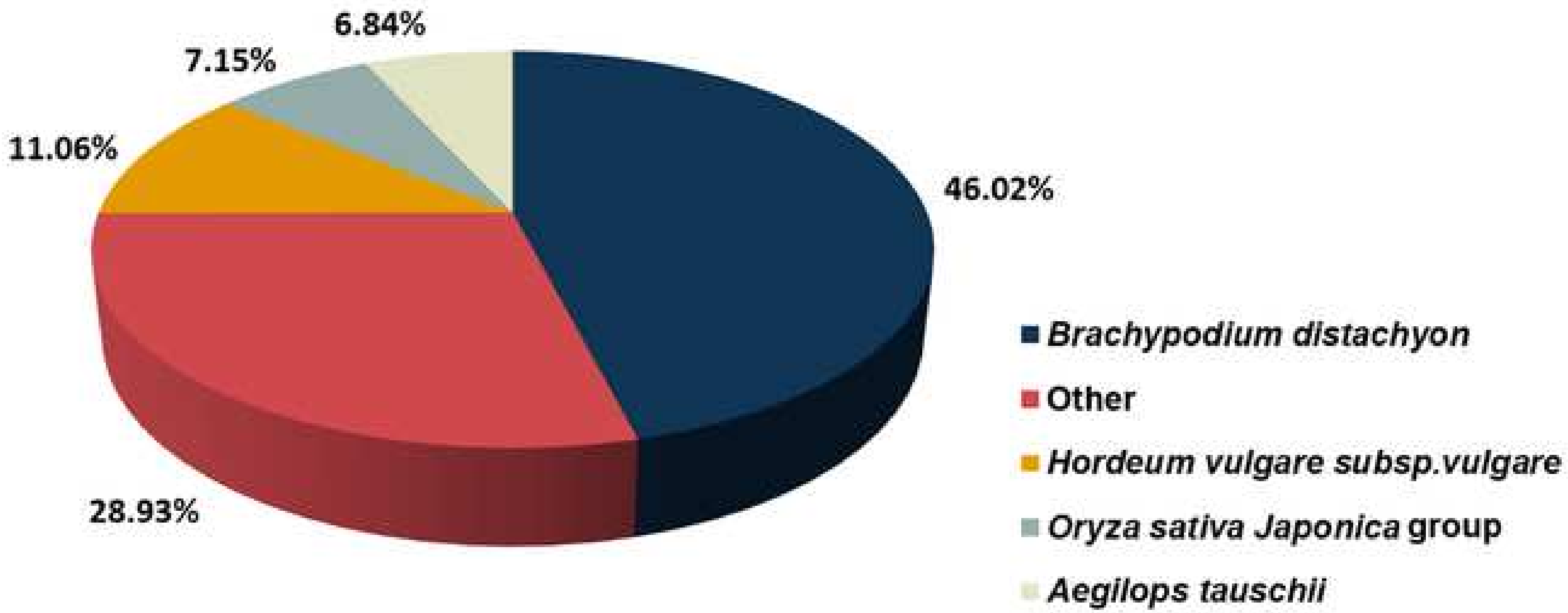

Figure

Click here to download Figure Fig2.tif

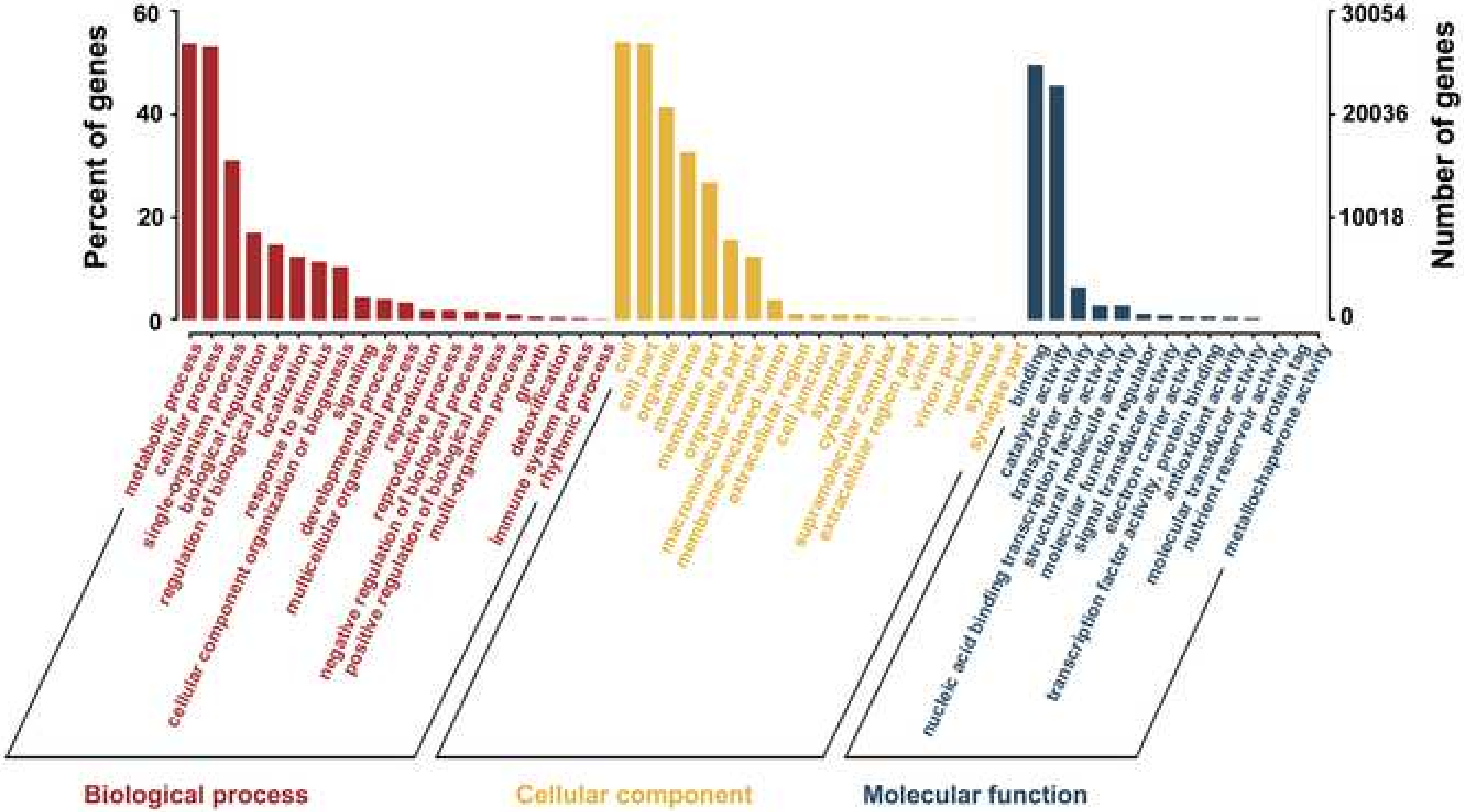

 Click here to download Figure Fig3.tif

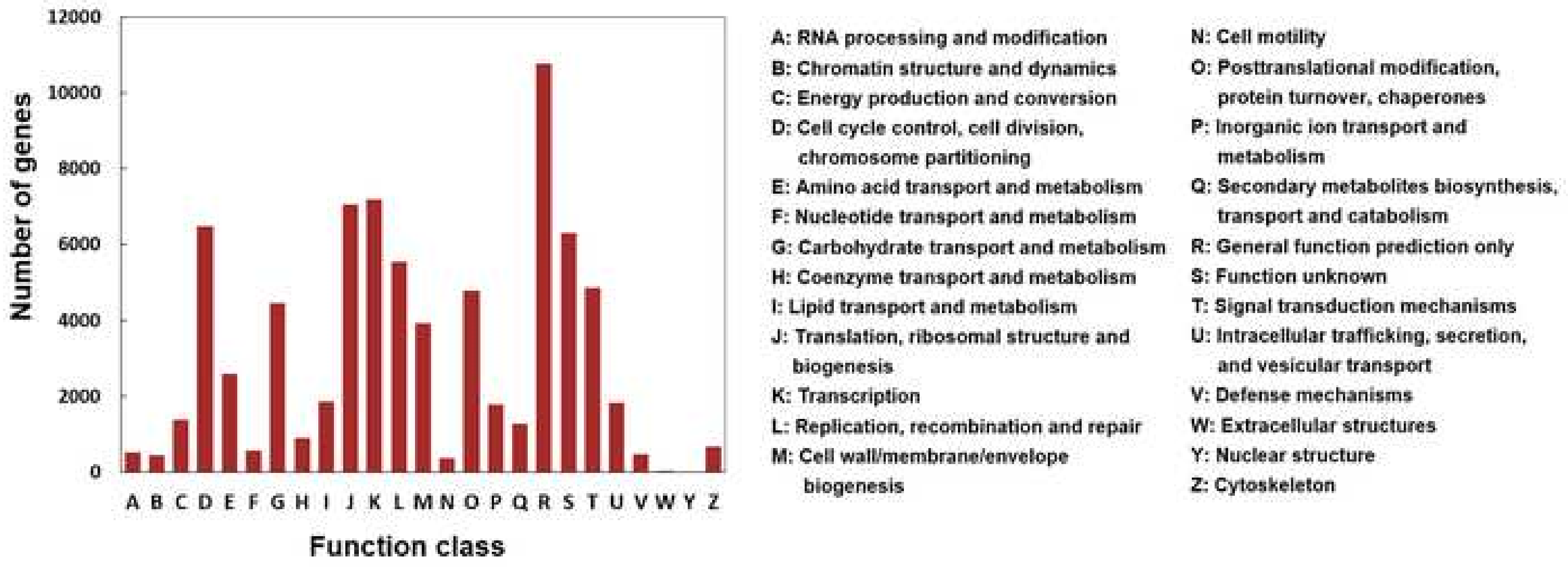

Click here to access/download
Supporting Information
S1_table.docx

Click here to access/download
Supporting Information
S2_table.xlsx

Click here to access/download
Supporting Information
a copy of manuscript.docx

Click here to access/download
Supporting Information
certificate.pdf
