# Supplementary material for: De novo assembly, annotation, marker discovery, and genetic diversity of the Stipa breviflora Griseb. (Poaceae) response to grazing
Source: PLoS One. 2020 Dec 22;15(12):e0244222. doi: 10.1371/journal.pone.0244222 (PMC7755183; doi:10.1371/journal.pone.0244222)
Supplement: Attachment — Submitted filename: Response to Revieweers.docx [file pone.0244222.s005.docx]

Dear reviewers:

Thank you for your comments on our manuscript entitled “*De novo* assembly, annotation, marker discovery, and genetic diversity of the *Stipa breviflora* Griseb. (Poacea) response to grazing” (ID: PONE-D-20-16248). Those comments are very helpful for revising and improving our paper, as well as the important guiding significance to our further researches. We have studied the comments carefully and made corrections which we hope meet with approval. The main corrections are in the manuscript and the responds to the reviewers’ comments are as follows (the replies are in blue font). The page number and line number written in black are based on the “Revised Manuscript with Track Changes. docx” file. The page number and line number written in blue are based on the “Manuscript. docx” file. (Because the submission system recoded the page number of the original manuscript we uploaded, please refer to the line number to locate the modification).

Responds to the reviewers’ comments:

Reviewer 1: The work done on the grass species *Stipa breviflora* does not justify the need of transcriptomics for genetic diversity against grazing. Better to include the merits of the study for justification of NGS analysis. There are other well adapted techniques for studying genetic diversity. Also grazing is not a big problem for plant science research which limits the productivity or greening of the lands across the world. In addition, how the results will be helpful for further research programs.

Response: thanks for your comment. We agree with your suggestion. Typically, NGS data set are very large (several gigabases to terabases), which provide us more information about organisms. As a major application of NGS, RNA-seq provides convenience for the genetic diversity analysis, because thousands of molecular markers can be obtained from one transcriptome sequencing. Therefore, we used RNA-seq not only to explore the changes of *S. breviflora* under grazing at the transcriptome level, but also for subsequent molecular marker development.

Page 5, line 90-96:

The next-generation sequencing (NGS) technology have provided an efficient way to generate an abundance of genomic data. Compare with traditional low-throughput EST (Expressed Sequence Tag) sequencing by Sanger technology, RNA-sequencing (RNA-seq) offers more transcripts for both markers development and gene discovery, helping us to explore the molecular mechanism of genetic diversity and microcosmic interaction in organisms [30].

For artificial grasslands, grazing effects are mainly productivity reduction and late greening. But for natural grasslands, grazing also affects plant diversity, species composition and primary production, ecosystem function, as well as soil properties in arid, semi-arid grassland. In China, grassland degradation is advancing over wide areas through overgrazing. Especially in desert steppe, the species composition is relatively simple, and the environment is relatively harsh, making it susceptible to climate change and human activities. Therefore, long-term overgrazing negatively affects the grassland ecosystem and cause economic loss.

Through RNA-seq, we found 686 differentially expressed genes (DEGs). Using SSRs mined from DEGs, we found the grazing-related genes showed a higher polymorphism under grazing. These gene may be useful for breeding and cultivation. In addition, we found grazing promotes genetic diversity of *S. breviflora*, which may guide the management and utilization of desert steppe. A certain level of grazing seems to be more beneficial than less or no grazing at all. We expressed our opinion in the discussion section and conclusion section. Thanks again for your suggestions.

Page 29, line 431-440:

Another important force driven genetic diversity changes is gene polymorphism. So, we deliberately mined SSRs derived from DEGs of *S. breviflora*. As a result, we found a higher genetic diversity in grazing populations. This result suggests that grazing promotes the polymorphism of genes with relevance to grazing. These polymorphisms may endue *S. breviflora* with more grazing tolerance. Under long-term grazing pressure, more individuals with grazing tolerance survive, resulting in the increase of genetic diversity. Therefore, a suitable grazing intensity seems to be more beneficial for maintaining genetic diversity.

Page 31, line 466-468:

For maintaining genetic diversity of *S. breviflora*, a suitable grazing intensity is needed in order to implement rational use and conservation of desert steppe.

Reviewer 2:

Comment 1: in page 2, line 26, add “,over grazing pressure”.

Response: thanks for your comment, we have already made the changes.

Page 2, line 25-27:

Grasslands, however, currently suffer from severe degradation induced by human activities, overgrazing pressure and climate change.

Comment 2: in page 2, line 37, replace “polymorphic” with “SSR”.

Response: thanks for your comment, we have already made the changes.

Page 2, line 37:

Based on transcriptome sequencing data, we developed eight SSR molecular markers and investigated the genetic diversity of *S. breviflora* in grazed and ungrazed sites.

Comment 3: in page 3-4, line 63-65, rewrite the sentence “Although faced with pressures derived from grazing and climate change, this species has great potential for future expansion”.

Response: thanks for your suggestion, we have already rewritten the sentence to make it easier to understand.

Page 3, line 62-64:

Although *S. breviflora* is vulnerable to climate change and grazing, it still has great potential for expansion and evolution via hybridization with other feather grasses [13, 17, 20].

Comment 4: in page 5, line 87. Which grasses?

Response: thanks for your comment, and we have added the species name in this sentence.

Page 4, line 82-86:

Smith et al. [28] investigated the fine-scale spatial genetic structure in *Bouteloua curtipendula var. caespitosa*, *Bouteloua gracilis* and *Poa ligularis* and observed no differences in average gene diversity between populations of each species.

Comment 5: in page 5, line 103-105. Delete the sentence “This technique has been applied to plants such as *Arabidopsis* species [31], *Salvia miltiorrhiza* [32], Zea mays [33], and *Cajanus cajan* (L.) [34].”

Response: thanks for your comment, we have deleted the extra sentence to make our manuscript more concise.

Comment 6: page 6, line 123. Delete “of *S. breviflora*”.

Response: thanks for your comment, and we have deleted the extra part you point out.

Comment 7: page 6, line 125. Delete “of *S. breviflora*”.

Response: thanks for your comment, and we have deleted the extra part you point out.

Comment 8: page 7, line 134, 135, 137. Provide information of study area, including area of sites, utilization of each site, and the animals grazing in each site.

Response: thanks for your comment. We added the detailed information of the study area.

Page 6, line 126-127:

The area of each site is more than 1 hm^2^.

For utilization of each site, and the animals grazing in each site, we added some detailed information in utilization term in Table 1.

Page 8, Table 1.

**Table 1. Information of the observed sites of *S. breviflora.***

| **Code** | **Position** | **Altitude(m)** | **Utilization** | **Plant community** |
| --- | --- | --- | --- | --- |
| 1 | 111.21°E, 41.13°N | 1629 | fenced without grazing | *S. breviflora* + *S. krylovii* |
| 2 | 111.26°E, 41.19°N | 1681 | fenced without grazing | *S. breviflora, + S. krylovii* + *Thymus mongolicus* |
| 3 | 112.41°E, 41.17°N | 1668 | fenced without grazing | *S. breviflora* + *S. krylovii* |
| 4 | 111.19°E, 41.11°N | 1647 | sheep grazing | *S. breviflora, + S. krylovii* + *Thymus mongolicus* |
| 5 | 111.29°E, 41.25°N | 1614 | sheep grazing | *S. breviflora* + *Agropyron cristatum* |
| 6 | 111.31°E, 41.29°N | 1573 | sheep grazing | *S. breviflora, + S. krylovii* + *Cleistogenes squarrosa* |

Comment 9: in page 9, line 149. In grazed site also, a few plants may have not been grazed, how the sampling was done, the plant selection was intentionally grazed ones, how it was identified. Proper care was taken that saliva or other parts of animal were not mixed with leaf samples which can give false results.

Response: thanks for your comments and reminders on sampling. we apologize for the unclear sampling description in the manuscript.

For RNA-seq, we chose the individuals in obviously grazed patches in grazed sites. On the contrary, we chose the individuals that had not suffered any damage in ungrazed populations. Because transcriptome is time-sensitive and sensitive to environment. Even small disturbance would affect the gene expression level. In addition, after the stress disappears, gene expression will quickly return to the original level. Thus, the individuals in the above-mentioned two states can accurately reflect the changes of gene expression.

Page 9, line 134-137:

In June 2016, we collected 33 samples at each site, with 3 samples for RNA-seq and 30 samples for genetic diversity analysis. For RNA-seq, we chose healthy and intact individuals in ungrazed sites, and chose individuals in obviously grazed patches in grazed sites.

For genetic diversity analysis, we selected the individuals that had not suffered any damage whether they are in grazed populations or ungrazed populations. Because genetic diversity refers to the variations of alleles in DNA sequence. As we all know that DNA sequence remains stable in a long period. Thus, even we chose intact individuals in grazing populations, they can represent genetic diversity under grazing. Besides, the individual we selected can only represent that they had not been damaged during the current growing season, but it did not mean that they have not been damaged by grazing in previous years, because *S. breviflora* is a perennial herb and the grazing sites have been subjected to long-term heavy grazing.

Plant height of *S. brebviflora* is about 20 cm, and the width of clump crown is about 25 cm. Although *S. breviflora* is a perennial herb, this species is relatively small. Therefore, sampling at 10 m intervals is consistent with the principle of population genetics.

Page 9, line 140-143:

When collecting samples for genetic diversity analysis, we followed the principle of population genetics, choosing the healthy and intact individuals that were at least 10 m apart in both grazed and ungrazed populations.

Before RNA extraction, the samples had already rinsed with RNase-free dd water to remove substance that may affect the accuracy of the experiment as much as possible.

Comment 10: in page 24, line 352. In highly inbreeding species with very low heterozygosity, how grazing can enhance genetic diversity? It is not clear. Authors should provide some explanation based on facts.

Response: we apologize for the confusion and appreciate your comments. More accurately, *S. breviflora* is a facultative selfing species. We corrected the inaccurate description about the reproduction of *S. breviflora* in our manuscript.

Page 29, line 419-420:

*S. breviflora* is a facultatively selfing species.

We believe that grazing promotes the pollen flow and seed flow of *S. breviflora*, and lead to a higher genetic diversity.

Page 28-29, line 417-431:

Gene flow, one of the important forces leads to improving genetic diversity of natural plant populations, occurs by the spread of pollen, seeds, spores, and other genetic materials. *S. breviflora* is a facultatively selfing species. We found that pollen flow may promote sexual reproduction of *S. breviflora*. As Table 9 indicates, He in grazing populations are significantly higher than ungrazed populations. Therefore, pollen-mediated gene flow plays an important role in maintaining genetic diversity of *S. breviflora* populations while stressed by grazing [62]. In general, grazing affects plant reproduction by defoliation. *S. breviflora*, however, can complete life circle under heavy grazing by increasing reproductive investment, which brings about maintaining even increasing seed germination rate [63-65]. Grazing transfers seeds through livestock migration and fosters seedling establishment, resulting in the enhancement of seed-mediated gene flow, and consequently increasing genetic diversity [62], though seed-mediated gene flow was not mentioned in the present study.

Therefore, our data showed a lower Ho than He, which is consistent with the reproduction characteristics of *S. breviflora*. In addition, we add a Student’s test and the results showed that He were significantly higher in grazing populations than in ungrazed populations. That means grazing promotes sexual reproduction rates and increases the genetic diversity of *S. breviflora.*

Comment 11: in page 25, line 355. Significant or not?

Response: thanks for your comment. We did a Students’ test, and added the results in the manuscript.

Page 15, line 220-222:

R version 3.6.3 was used to perform Student’s test to explore the relations of genetic parameters between grazed and ungrazed populations.

Page 24, line 338-340:

As shown in Table 9, both Shannon’s information index (I) and expected heterozygosity (He) in grazing populations are higher significantly than that in ungrazed populations (p<0.05).

Page 25-26, line 349-356:

**Table 9. Student’s test of genetic parameters between ungrazed and grazing populations.**

|  | **Na^a^** | **Ne^b^** | **Ho^c^** | **He^d^** | **I^e^** |
| --- | --- | --- | --- | --- | --- |
| df | 4 | | | | |
| P value | 0.131 | 0.196 | 0.423 | 0.019* | 0.015* |

^a^Na: observed number of alleles.

^b^Ne: effective number of alleles.

^c^Ho: observed heterozygosity.

^d^He: expected heterozygosity.

^e^I: Shannon’s information index.

*p<0.05

Comment 12: in page 25, Table 8. Observed heterozygosity is more in ungrazed population 1 and 3 as compared to grazed population 4, 5, 6. Needs explanation. Except for population 2, not much difference is seen in rest 5 populations. Interpretation of data needs to look into this fact.

Response: we did a Student’s test to examine the significance between grazed and ungrazed populations. Even pop1 and 3 has a higher Ho, there is no significant difference between grazed and ungrazed populations. We think this is due to randomly sampling.

For PPB (percentage of polymorphic bands), we checked the original data. In pop 2, there is only one genotype in CL966.Contig15, which means that there is no polymorphic bands in pop2 at CL966.Contig15. That is why the PPB in pop2 is 87.5%.

Comment 13: in page 26, line 384-388. Too broad assumption, supporting data needed. The upregulation of genes are common response to cutting, defoliation or grazing. Does it mean change in genetic diversity? It is usual that genes get activated due to some injury or climatic shock but the genes are again at same level of regulation once that shock is overcome, It needs further clarification how it is enhancing genetic diversity?

Response: thanks for your comment. We apologize for the unclear statement in the discussion section.

We agree with your comment. The upregulation of genes are common response to cutting, defoliation or grazing. The SSRs we used were mined from DEGs. In grazing populations, we observed a higher genetic diversity. That means the DEGs have more polymorphisms under grazing. Therefore, grazing could enhance genetic diversity of *S. breviflora*.

Page 29, line 431-435:

Another important force driven genetic diversity changes is gene polymorphism. So, we deliberately mined SSRs derived from DEGs of *S. breviflora*. As a result, we found a higher genetic diversity in grazing populations. This result suggests that grazing promotes the polymorphism of genes with relevance to grazing.

Comment 14: in page 28, line 426-428. Similar studies of upregulation of such genes have been reported by other authors in different plants due to injury or wounding. How in the present study it is related specifically to grazing?? It can be a simple sounding carried out by any activity. The same sets of genes will again be at normal level when the wounding shock is overcome after some time. So it does not create new genetic diversity. It needs response form authors.

Response: thank you for your constructive comment. Grazing and other types of damage can cause upregulation of genes related to JA biosynthesis. We list genes related to JA because the major effect of grazing on plants is wounding and the top enriched KEGG pathway is about JA synthesis. In addition, grazing is a complex process that contains wounding, defoliation, and saliva deposition. It is difficult to distinguish the effects that caused only by grazing but not other factors. In the present study, we regarded grazing as a whole process and did not distinguish the genes only induced by grazing. We also explained in the manuscript.

Page 27, line 375-376:

As a main effect of grazing on herbage, wounding threat is inevitable and occurs over the entire life [35].

Page 26, line 381-384:

For *S. breviflora*, genes such as phospholipase A1 (*PLA_1_*) and 13-lipoxygenase (13-*LOX*), which are know to be involved in the synthesis of JA, were upregulated. For other plants, leaf wounding response were also related to JA synthesis.

By comparing with the research that simulates the effects of grazing on transcriptome, we also found a gene that was only induced by grazing. We introduced that gene in our manuscript.

Page 27-28, line 391-397:

Herbivore grazing, however, is a joint process that includes wounding, defoliation, and saliva deposition. Chen et al. [52] used rice to study grazing-induced pathway, and found the expression level of 1,3-beta-glucosidase increased. Our study showed the coincident result. We observed genes related to beta-glucosidase, which were confirmed to be involved in cell wall construction, modification, expansion, and maintenance, were upregulated.

Comment 15: in page 30, line 463. The results could have been same if damage to plants would have been caused due to mechanical or manual harvesting?

Response: thanks for your thought-provoking comment. Because wounding is a major effect of grazing on plants, there must be some genes shared between grazing and other types of damages. But in our study, the 686 DEGs also contain genes that only induced by grazing. We regarded grazing as a whole process and did not design an experiment to distinguish grazing damage from other type of damages. Compared with the study of Chen et al., we also found genes that only induced by grazing. Therefore, the results caused by grazing and other damages are not exactly same.

Page 27, line 389-391:

*PLA_1_* and *13-LOX* are shared by grazing and other wounding response in different species, indicating that plants have some common mechanisms in defense response.

Comment 16: in page 31, line 483, 487. Changes in gene sequences are not justified comments. It can at best be changes in gene expression in response to stress. How DNA sequences have changed to resist grazing pressure?

Response: thanks for your comment. We apologize for our confusing expression. What we originally wanted to express was: In response to grazing, *S. breviflora* not only develops resistance capabilities by regulating gene expression but also exhibits polymorphisms in DNA sequences. That expression was deleted, because we reorganized the discussion section.

Comment 17: in page 31, line 492-493. Delete the sentence “this phenomenon is supported in many plants, such as *E. nutans* and *S. purpurea*”.

Response: thanks for your comment, we have deleted the sentence.

Comment 18: in page 33, line 519. It is quite common that animals such as birds, insects, herbivores or even carnivores help in seed dispersal. Normally animals prefer the plants which are in vegetative stage and avoid the mature plants where seed setting has occurred, unless it is very heavily grazed.

Response: thanks for your comment. We agree your suggestion. We also looked through the related literature and found that *S. breviflora* could complete its life circle by increasing reproductive investment under grazing. Seed production of *S. breviflora* showed no significant difference between heavy grazed and ungrazed sites. We believe that this is a special strategy for *S. breviflora* to adapt to grazing over a long period of time. Therefore, we answered your question in the manuscript to make our opinions more rigorous.

Page 29, line 424-427:

In general, grazing affects plant reproduction by defoliation. *S. breviflora*, however, can complete life circle under heavy grazing by increasing reproductive investment, which brings about maintaining even increasing seed germination rate [63-65].

Comment 19: in page 33, line 528. In a self-pollinated species with very low heterozygosity how seed dispersal will increase the diversity needs more explanation.

Response: thanks for your comment. We apologize for our inaccurate expression about the reproduction strategy of *S. breviflora*. Accurately, *S. breviflora* is a facultative selfing species. That is why pollen flow also contribute to the reproduction of *S. breviflora*.

Page 29, line 419-420:

*S. breviflora* is a facultatively selfing species.

In addition, our data showed a higher He in grazing populations than in ungrazed populations. This may be due to grazing promote the pollen flow, and increase the ratio of sexual reproduction, and therefore caused a higher genetic diversity.

Page 29, line 420-424:

We found that pollen flow may promote sexual reproduction of *S. breviflora*. As Table 9 indicates, He in grazing populations are significantly higher than ungrazed populations. Therefore, pollen-mediated gene flow plays an important role in maintaining genetic diversity of *S. breviflora* populations while stressed by grazing [62].

Comment 20: in page 33, line 528. Accessing?

Response: thanks for your comment. We rephrased the sentence to make it easier to understand.

Page 30, line 442-443:

SSR system is more efficient for genetic analysis than the ISSR system [66].

Comment 21: in page 33, line 532. The presumption needs support of data. Not likely? How trampling can cause increase in sexual reproduction? Clonal growth can be reduced by trampling. But damage to main tiller causes more branch and tiller proliferation.

Response: thanks for your comment. We agree with your comment. We have deleted that sentence.

Comment 22: in page 33, line 534. Is it effect of grazing by animals or simple defoliation or cut and carry system could have caused similar effects. There are certain phenomenon associated with the defoliation or cutting the plants manually/ mechanically or grazing, the growth hormones, wound healing genes, will be activated.

Response: thanks for your comment, we agree with your comment. Grazing by animals or other types of wounding such as defoliation and cutting could cause similar effects. Because wounding is the major effect that caused by grazing, there must be some similar effects between grazing and other types of wounding. That is why the pathway related to JA synthesis ranked top among the pathways annotated by KEGG database. However, the “effect” in the manuscript refers to all the effects caused by grazing, and we did not distinguish the common and distinct effects for *S. breviflora* response to grazing compared to other types of mechanical wounding.

Comment 23: in page 34, line 548. How grazing increases biodiversity is not clear?? Grazing by animals can increase seed dispersal, only if grazing is continued beyond maturation stage. Usually grazing is done at vegetative stage and if grazing intensity is high it results in regeneration associated with delayed or no flowering. Animals can help in seed dispersal only if they are allowed to move in seed maturation stage.

Response: thanks for your constructive comment. Grazing dose have an impact on plant reproduction, especially at vegetation stage. However, for *S. breviflora*, researchers found that *S. breviflora* adapted to heavy grazing by reducing height and increasing reproductive input and there is no significant differences in seed production between heavy grazing and no grazing conditions. Based on this special strategy, we assumed that grazing promote seed flow of *S. breviflora* and lead to an increased genetic diversity.

Page 29, line 424-431:

In general, grazing affects plant reproduction by defoliation. *S. breviflora*, however, can complete life circle under heavy grazing by increasing reproductive investment, which brings about maintaining even increasing seed germination rate [63-65]. Grazing transfers seeds through livestock migration and fosters seedling establishment, resulting in the enhancement of seed-mediated gene flow, and consequently increasing genetic diversity [62], though seed-mediated gene flow was not mentioned in the present study.

Comment 24: in page 34, line 558. The study would have been more informative if another aspect of manual defoliation been added and transcriptome analysis carried out in three groups, ungrazed, grazed and manually cut. The difference would have in that case highlighted the grazing demand. Another aspects of 3 stage study, one ungrazed field, second 5-10 days or during grazing and third one month after stopping the grazing would have given clear picture whether these upregulation or down regulation of genes are stable or just transient phenomenon due to stress.

Response: thanks for your constructive suggestions. We deeply agree with your opinion. Due to time constraints (we need to wait until June of next year to perform this experiment again), unfortunately we can not carry out this experiment according to your suggestions. But your comments are instructive and provide great ideas for our further work. Thanks again for your thought-provoking suggestions.

Reviewer 3:

Comment 1: in page 2, line 40-41, delete “This study enriches the knowledge of the genetic background of *S. brevifora*”.

Response: thanks for your suggestion, we have deleted this sentence.

Comment 2: in page 3, line 45-47, delete “include meadow grasslands, typical grasslands, desert grasslands, and alpine grasslands. The grasslands in China”.

Response: thanks for your careful modification, we have deleted the extra part and rewrote the sentence to made it more concise.

Page 3, line 45-47:

Chinese grasslands are diverse and constitute the third largest grassland area worldwide, covering 41.7% of the country’s territory and stretching from northern China to the Qinghai-Tibetan Plateau [1-3].

Comment 3: in page 3, line 49, add reference “sPlot –A new tool for global vegetation analyses”

Response: thanks for the reference you supplied to us, it makes our manuscript more informative and we have already added the reference.

Page 3, line 47

Comment 4: in page 3, line 55, add “(Poaceae)” after “species”.

Response: thanks for your comments on the rules for writing species names, it helped a lot. We will pay more attention to this issue in the manuscript, and we followed your suggestion.

Page 3, line 54:

*Stipa* species (Poaceae) are the most conspicuous grassland plants distributed throughout the Eurasian Steppe and are established as zonal vegetation in the Inner Mongolia grassland.

Comment 5: in page 3, line 58, add reference “Freitag 1985, Notes royal Bot Garden 42; Wu and Phillips 2006, Flora of China; Nobis et al. 2016, Phytotaxa 267(1); 2020, Annales Misouri Bot gard 105”.

Response: thanks for the references you supplied to us. We did ignore some important references about the background of *Stipa* species (Poaceae), and we have already added the references.

Page 3, line 56-57.

Comment 6: in page 3, line 58, delete “(Poaceae)”.

Response: thanks for your comments and we have already made the change.

Comment 7: in page 3, line 58, replace “a” with “one of”.

Response: thanks for your comment. We have replaced “a” with “one of the”.

Page 3, line 57:

*S. breviflora* Griseb. is one of the dominant species and valuable forage resource in the desert steppe and is characterized by palatability, rich nutrient content, and early greening.

Comment 8: in page 3, line 63, add more suitable references, eg.: Lu & Wu 1996, Acta Phytotax Sin 34; Nobis et al. 2019, Perpectives in plant ecol evil syst. 39; Baiakhmetov et al 2020 Sci Reports.

Response: thanks for your comment and we have added the references.

Page 3, line 62.

Comment 9: in page 4, line 65, add reference Baiakhmetov et al. 2020 Sci Rep and Nobis et al. 2020.

Response: thanks for your comment. We added the references and the sentence you modified.

Page 3, line 62-64:

Although *S. breviflora* is vulnerable to climate change and grazing, it still has great potential for expansion and evolution via hybridization with other feather grasses [13, 17, 20].

Comment 10: in page 4, line 72, add references, eg.: Klichowska et al 2020. Ecological Indicators 111; Klichowska et al 2019, Applied Soil Ecology 144.

Response: thanks for your comment, we have added the references.

Page 4, line 69.

Comment 11: in page 4, line 76, change “*Stipa* genus” to “genus *Stipa*”.

Response: thanks for your comment, we switched the position of the two words.

Page 4, line 73:

In the genus *Stipa*, moderated grazing was found to promote the genetic diversity of S. grandis and *S. krylovii.*

Comment 12: in page 4, line 85, write the authors’ name.

Response: thanks for your comment. We apologize for the confusion we made. We use the sentence “researchers found that grazing had no significant effect on plant genetic diversity.” to summarize the research situation in general, and does not mean to refer to any specific researcher. Thus, we use a different expression to avoid ambiguity.

Page 4, line 81-82:

In some cases, grazing had no significant effect on plant genetic diversity.

Comment 13: in page 6, line 115. The expression “grow significantly smaller” is not clear.

Response: thanks for your comments, and we have changed another expression.

Page 5, line 106:

For *S. grandis*, individuals subjected to grazing develop smaller size than those not subjected to grazing, and the expression of wound-, drought-, and defense-related genes is altered [35].

Comment 14: in page 23, line 326. “most allele with 5” is not clear.

Response: thanks for your comment. We apologize for our unclear expression in the manuscript.

Page 23, line 313-314:

Among these primers, CL966.Contig12 and CL.5285.Contig5 produced the largest number of alleles with 5.

Comment 15: in page 23, line 328-330. Move the sentence “suggesting lower polymorphism than the results by Ren et al., who reported 6 alleles per primer.” to discussion.

Response: thanks for your comment. Because we reorganized the discussion section, this sentence was deleted.

Comment 16: in page 27, line 389-390. In my opinion this subchapter is definitely too long (in comparison to the results) and over-discussed. It should be definitely shortened.

Response: thanks for your comment. We have already rephrased and shortened this section.

Page 27-28, line-375-407.

Comment 17: in page 31, line 492-493. Provide genera names please.

Response: thanks for your suggestion. Because we have reorganized this discussion section, this sentence was deleted from the manuscript. But your comments helped us a lot in writing in a formal way, and we will pay more attention to this issue in future writing.

Comment 18: in page 32, line 496. This is not true. *Stipa breviflora* is not obligatory self-pollinator. Flowers of this species can be pollinated also by pollen of other individuals od this taxon. That is why, there are many hybridisations and introgression events in Stipa, also with share of *S. berviflora* (see Baiakhmetov et al. 2020 Sci Rep).

Response: thanks for your professional comments. Your comments not only help increase the accuracy of our statement, but also make our discussion section more convincing. We apologize for our negligence, and we have already corrected our mistake.

Page 29, line 419-420:

*S. breviflora* is a facultatively selfing species.

Comment 19: in page 33, line 526. see also in *Stipa pennata* - Klichowska et al. 2018 Molecular Biology Reports 45(4).

Response: thanks for your suggestion, we have added this reference in the abstract section.

Page 5, line 98-99:

Klichowska et al. [32] mined ten microsatellite markers of *Stipa pennata* using Illumina high-throughput.

Comment 20: in page 33, line 534-535. Delete the sentence “The aim of the present study was to evaluate the effects of grazing on genetic diversity”.

Response: thanks for your comment, and we have already deleted this sentence.

Comment 21, in page 33, line 535-536. Rephrase the sentence “The grazing intensity was not considered in our study. However, grazing intensity has been taken into account in other study”.

Response: thanks for your comment. We have already rephrased these sentences.

Page 30, line 446-447:

In addition, grazing intensity has also been taken into account in other studies.

Comment 22: in page 35, line 561. Replace “codominates” with “occur”.

Response: thanks for your suggestion. We have already finished the replacement.

Comment 23: in page 35, line 563. Use the plural form of “steppe”.

Response: thanks for your comment. we have made the change.

Comment 24: in page 35, line 570. This is very general conclusion which is more suitable for abstract. However, it would be much valuable if you could explain to readers how the study can help in conservation and restoration (having in mind that grazing impact on genetic diversity!). The second question is that, whether grazing of steppe vegetation is regard as threat or rather not grazing should be regarded as such one. Steppes were always under such animal pression, so maybe even more important problem in this case could be impact of not grazing that could influence on less genetic diversity and probability of extinction.

Response: thanks for your thought-provoking comments. We explained this question in the discussion section.

Page 29, line 431-440:

Another important force driven genetic diversity changes is gene polymorphism. So, we deliberately mined SSRs derived from DEGs of *S. breviflora*. As a result, we found a higher genetic diversity in grazing populations. This result suggests that grazing promotes the polymorphism of genes with relevance to grazing. These polymorphisms may endue *S. breviflora* with more grazing tolerance. Under long-term grazing pressure, more individuals with grazing tolerance survive, resulting in the increase of genetic diversity. Therefore, a suitable grazing intensity seems to be more beneficial for maintaining genetic diversity.

We re-expressed this sentence in conclusion section.

Page 31, line 466-468:

For maintaining genetic diversity of *S. breviflora*, a suitable grazing intensity is needed in order to implement rational use and conservation of desert steppe.

Because of the long-term association of grazing animals and steppe, it is not surprising that plants have evolved mechanisms to adapt to grazing. That is why *S. breviflora* can remain stable in the community even under grazing. Without grazing, steppe would not be the same as we see now. Long-term overgrazing is bound to reduce the genetic diversity of plants. Long-term nongrazing is not necessarily beneficial to steppe. Therefore, suitable grazing intensity may be a good strategy for steppe conservation, which is in line with “intermediate disturbance hypothesis”. In addition, time scale needs to be considered. There are negative effects on *S. breviflora* conservation if grazing or prohibit grazing last too long.

Comment 25: figure and table captions are unavailable or there are no such captions in the manuscript.

Response: thanks for your comment. We had checked the captions of every figure and table. We followed the requirement of the journal: figure captions area inserted immediately after the first paragraph in which the figure is cited, and the figure files are uploaded separately. Tables are inserted immediately after the first paragraph in which they are cited. Thanks again for all your thoughtful suggestions and it really helped us a lot.
