# Supplementary material for: De novo assembly, annotation, marker discovery, and genetic diversity of the Stipa breviflora Griseb. (Poaceae) response to grazing
Source: PLoS One. 2020 Dec 22;15(12):e0244222. doi: 10.1371/journal.pone.0244222 (PMC7755183; doi:10.1371/journal.pone.0244222)
Supplement: Attachment — Submitted filename: Response to Reviewers.docx [file pone.0244222.s006.docx]

Dear editor：

We would like to thank you for the opportunity to revise our manuscript entitled “*De novo* assembly, annotation, marker discovery, and genetic diversity of the *Stipa breviflora* Griseb. (Poacea) response to grazing” (ID: PONE-D-20-16248R1). We are grateful to you for constructive comments and thoughtful suggestions.

We have analyzed all the comments carefully and modified our manuscript to address editor’s concern and accommodate their suggestions. The corrections are in the manuscript and the response to the editor’s comments are as follows (the replies are in blue font). The line number written **in black** are based on the “Revised Manuscript with Track Changes. docx” file. The line number written **in blue** are based on the “Manuscript. docx” file.

Again, we thank editor in chief, academic editor and another two reviewers for extremely constructive suggestions that have improved the quality of our manuscript. Please let us know if you have any questions regarding the manuscript. We are looking forward to hearing from you.

Response to the editor’s comments:

Comment 1: L91-92: "Compare with traditional..." - wrong tense. L95-96: Expression "microcosmic interaction in organisms" is rather picturesque, but fails to refer to scientific facts.

Response: thanks for your comment, we have already modified the tense and deleted the wrong expression “microcosmic interaction in organisms”.

Line 91-95:

Compared with traditional low-throughput EST (Expressed Sequence Tag) sequencing by Sanger technology, RNA-sequencing (RNA-seq) offers more transcripts for both marker development and gene discovery, helping us to explore the molecular mechanism of genetic diversity [30].

Comment 2: L440-444: Sentences: "This result suggests that grazing promotes the polymorphism of genes with relevance to grazing." and "Under long-term grazing pressure, more individuals with grazing tolerance survive, resulting in the increase of genetic diversity." cannot be true, but just the opposite. If grazing tolerance has the genetic background, then narrowing variation of a phenetic character would result in increasing homozygosity of the genes involved in such a trait, which through generations leads to deletion of rare alleles, and finally to the decrease in genetic variations (of the genes under the selective pressure). Authors should further elaborate the presented fact and to bring their stance throughout the text.

Response: thanks for your comment. We agree with your suggestions and apologize for our misleading expression. Obviously, our expression is appropriate. In order to make our manuscript more rigorous, we deleted the sentence “Under long-term grazing pressure, more individuals with grazing tolerance survive, resulting in the increase of genetic diversity. Therefore, a suitable grazing intensity seems to be more beneficial for maintaining genetic diversity.”.

Because if we discuss the selection effects of grazing pressure on related genes, grazing intensity is an indispensable factor. But in our study, grazing intensity is not in our consideration. So based on our current results, we could not explain the effects of selection force on genetic diversity clearly. That is why we deleted these sentences. But your comment is very helpful for us to deeply understand the nature of genetic diversity an provides good idea for our further study.

The other reason is to avoid duplication. As you mentioned that strong selective pressure may cause gene homozygosity, and ultimately lead to decreased genetic diversity, which is consistent with many researches. For example, in Peng et al. study, the genetic diversity of both *S. grandis* and *S. krylovii* populations show the highest value under moderate grazing intensity, whereas the lowest under the heavy grazing. Shan et al. also find that the population under light grazing has highest genetic diversity. Therefore, we think moderate or light grazing intensity seem to be beneficial for maintaining genetic diversity of *Stipa* grasslands. In addition, the effects of grazing on genetic diversity varies in different plants. We have also elaborated the phenomenon in line 444-453 and cited these references in the manuscript.

Comment 3: L142: "principle of population genetics" does not describe how to gather the samples for a study. That may be "General Principles of Good Sampling Practice".

Response: thanks for your comment, we have already deleted the extra expression “principle of population genetics” and rewritten the sentence.

Line 140-142:

For genetic diversity analysis, we chose the healthy and intact individuals that were at least 10 m apart in both grazed and ungrazed populations.

Comment 4: L386-387: "For other plants, leaf wounding response were also related to JA synthesis." - please provide references here.

Response: thanks for your comment. We have added the references.

Line 385-386:

For other plants, leaf wounding response were also related to JA synthesis [50, 51].

Comment 5: L397: Increment of 1,3-beta-glucosidase expression under the mechanical wounding should be further elaborated and referenced.

Response: thanks for your comment. We have already added reference and further elaborated the gene function.

Line 396-399:

This gene was reported to be associated with resistance in plant, cell wall construction and modification [55,56]. In our study, gene related to beta-glucosidase was upregulated under grazing, conferring resistance on *S. breviflora*.

Comment 6: L316: "number of alleles with 5" - wrong formulation.

Response: thanks for you comment, we have already rephrased the sentence. Thanks again for your thoughtful suggestions and it really helped us a lot.

Line 315-316:

Among these primers, CL966.Contig12 and CL.5285.Contig5 produced the largest number of alleles, i.e. five alleles.
